# Epigenetic adaptations of the masticatory mucosa to periodontal inflammation

**DOI:** 10.1186/s13148-021-01190-7

**Published:** 2021-11-03

**Authors:** Gesa M. Richter, Jochen Kruppa, H. Gencay Keceli, Emel Tuğba Ataman-Duruel, Christian Graetz, Nicole Pischon, Gunar Wagner, Carsten Rendenbach, Yvonne Jockel-Schneider, Orlando Martins, Corinna Bruckmann, Ingmar Staufenbiel, Andre Franke, Rahime M. Nohutcu, Søren Jepsen, Henrik Dommisch, Arne S. Schaefer

**Affiliations:** 1 Department of Periodontology and Synoptic Dentistry, Oral Medicine and Oral Surgery, Institute for Dental and Craniofacial Sciences, Charité – Universitätsmedizin Berlin, Freie Universität Berlin, Humboldt-Universität Zu Berlin, and Berlin Institute of Health, Aßmannshauser Str. 4-6, 14197 Berlin, Germany; 2 Institute of Medical Informatics, Charité – Universitätsmedizin Berlin, Freie Universität Berlin, Humboldt-Universität Zu Berlin, and Berlin Institute of Health, Charitéplatz 1, 10117 Berlin, Germany; 3 Periodontology Department, Faculty of Dentistry, Hacettepe University, 06230 Sihhiye/Altindag/Ankara, Turkey; 4 Clinic of Conservative Dentistry and Periodontology, University Medical Center Schleswig-Holstein, Arnold-Heller-Straße 3, 24105 Kiel, Germany; 5 Private Practice, Karl-Marx-Straße 24, 12529 Schönefeld, Germany; 6 Department of Restorative Dentistry and Periodontology, University Medical Center Leipzig, 04103 Leipzig, Germany; 7 Department of Oral and Maxillofacial Surgery, Charité - Universitätsmedizin Berlin, Freie Universität Berlin, Humboldt-Universität Zu Berlin, and Berlin Institute of Health, Augustenburger Platz 1, 13353 Berlin, Germany; 8 Department of Periodontology, Clinic of Preventive Dentistry and Periodontology, University Medical Center of the Julius-Maximilians-University, Pleicherwall, 97070 Würzburg, Germany; 9 Institute of Periodontology, Institute of Medicine and Oral Surgery, Dentistry Department, Faculty of Medicine, University of Coimbra, Av. Bissaya Barreto, Bloco de Celas, 3000-075 Coimbra, Portugal; 10 Department of Conservative Dentistry and Periodontology, Medical University Vienna, School of Dentistry, Sensengasse 2a, 1090 Vienna, Austria; 11 Department of Conservative Dentistry, Periodontology & Preventive Dentistry, School of Dentistry, Hannover Medical School (MHH), Carl-Neuberg-Str. 1, 30625 Hannover, Germany; 12 Institute of Clinical Molecular Biology, Christian-Albrechts-University, Rosalind-Franklin-Straße 12, 24105 Kiel, Germany; 13 Department of Periodontology, Operative and Preventive Dentistry, University of Bonn, Welschnonnenstraße 17, 53111 Bonn, Germany

**Keywords:** EWAS, Methylation, Periodontitis, Gingiva, Inflammation, Cell type deconvolution, *ROBO2*, *PTP4A3*

## Abstract

**Background:**

In mucosal barrier interfaces, flexible responses of gene expression to long-term environmental changes allow adaptation and fine-tuning for the balance of host defense and uncontrolled not-resolving inflammation. Epigenetic modifications of the chromatin confer plasticity to the genetic information and give insight into how tissues use the genetic information to adapt to environmental factors. The oral mucosa is particularly exposed to environmental stressors such as a variable microbiota. Likewise, persistent oral inflammation is the most important intrinsic risk factor for the oral inflammatory disease periodontitis and has strong potential to alter DNA-methylation patterns. The aim of the current study was to identify epigenetic changes of the oral masticatory mucosa in response to long-term inflammation that resulted in periodontitis.

**Methods and results:**

Genome-wide CpG methylation of both inflamed and clinically uninflamed solid gingival tissue biopsies of 60 periodontitis cases was analyzed using the Infinium MethylationEPIC BeadChip. We validated and performed cell-type deconvolution for infiltrated immune cells using the EpiDish algorithm. Effect sizes of DMPs in gingival epithelial and fibroblast cells were estimated and adjusted for confounding factors using our recently developed “intercept-method”. In the current EWAS, we identified various genes that showed significantly different methylation between periodontitis-inflamed and uninflamed oral mucosa in periodontitis patients. The strongest differences were observed for genes with roles in wound healing (*ROBO2, PTP4A3*), cell adhesion (*LPXN*) and innate immune response (*CCL26, DNAJC1*, *BPI*). Enrichment analyses implied a role of epigenetic changes for vesicle trafficking gene sets.

**Conclusions:**

Our results imply specific adaptations of the oral mucosa to a persistent inflammatory environment that involve wound repair, barrier integrity, and innate immune defense.

**Supplementary Information:**

The online version contains supplementary material available at 10.1186/s13148-021-01190-7.

## Background

Genetic studies of chronic inflammatory diseases focused primarily on identifying DNA variants (e.g., single-nucleotide polymorphisms, SNPs) that confer disease risk through genome-wide association studies (GWASs; see [1, 2]). More recently, studies have also examined differences between patients and controls in patterns of DNA methylation (see [3, 4]). Methylation of cytosine is the most common modification of DNA that occurs at CpG dinucleotides (cytosine followed by guanine). It typically causes chromatin condensation and disruption of interactions between DNA and transcription factors, which are associated with transcriptional regulation [5]. Such epigenetic modifications of the chromatin confer plasticity to the genome and enable flexible and reversible responses of the genetic information to environmental challenges, allowing long-term adaptation and fine-tuning of gene expression levels [6]. Correspondingly, epigenetic changes give direct insight into the genetic information that the tissue uses to adapt to environmental factors. A major focus of research into the cellular mechanisms that underlie chronic disease is the tissue-environment interface. Particularly in the mucosa, a balance must be maintained between host defense and uncontrolled, not-resolving inflammation [7, 8]. Maintaining tissue integrity requires continuous adaptations of immune responses, barrier function and regeneration to a changing external and internal environment. It is considered that the development of adverse immune reactions may be associated with these challenges.

The oral masticatory mucosa locates to the alveolus/tooth complex and the hard palate and comprises all parts of the oral mucosa that are involved in chewing. Its position at the entrance to the gastrointestinal tract and respiratory systems entails its direct exposition to a diverse microbiota and various other environmental stressors such as tobacco smoke. If the mucosal barrier is impaired, pathogens can invade into subjacent tissue layers and promote periodontal inflammation. Persisting inflammation causes gingival bleeding and leads to the loss of connective tissue and alveolar bone with subsequent tooth loss, which determines the clinical characteristics of the common oral inflammatory disease periodontitis. In the etiology of periodontitis, persistent gingival inflammation is the most important risk factor [9], followed by smoking [10, 11]. Inflammation and smoking show strong effects in altering DNA-methylation patterns. This implies that in relation to the masticatory mucosa both can be considered as ‘environmental variables’ with a causal role for the onset and progression of periodontitis.

Recently, an epigenome-wide association study (EWAS) to investigate the specific methylation and expression patterns of the healthy masticatory mucosa in the context of cigarette smoke exposure was performed by our group [12]. In this study, differentially methylated positions (DMPs) were re-discovered to be associated with smoking status at a genome-wide significance level within the genes *CYP1B1* (cytochrome P450 family 1 subfamily B member 1) and *AHRR* (aryl-hydrocarbon receptor repressor). Several EWAS have identified these associations for cells of the alveolar [13] and buccal mucosa [14] before, putting emphasis on the role of epigenetic adaptations of the activity of these genes in barrier tissues that are long-term exposed to tobacco smoke metabolites. Some of these associations were restricted to solid epithelial tissues and were not found in blood, indicating the tissue-specificity of CpG methylation patterns relating to tissue function.

To date, few studies were published on differential DNA methylation in affected solid tissues in the course of inflammatory disease, where differences in cell-type composition are disease-immanent, as cell-type deconvolution algorithms still lack specific reference panels for many cell types. EpiDish provides an accurate reference dataset for generic epithelial tissue [15]. In the present study, we aim to confirm its suitability for the detection of cells specific for gingival tissue and employ it for cell-type deconvolution of our data. As differences in cell-type composition represent a major confounding factor in EWAS of inflammatory diseases, they also hinder the interpretation of effect sizes in affected tissue, which may be crucial for evaluating the relevance of significant associations. By applying our recently published “intercept-method” for the inference of effect sizes adjusted for confounding factors [16] to our data, we provide adapted estimations of the effect sizes at DMPs, which allows us to shed light on the molecular mechanisms altered in the etiology of periodontitis.

The aim of the current study was to investigate epigenetic changes in the gingiva in the course of long-term inflammation that resulted in periodontitis. To this end, the methylation patterns of inflamed and clinically uninflamed gingival tissue biopsies of 60 periodontitis cases were analyzed. To our knowledge, this is the first EWAS that investigated genome-wide epigenetic changes in solid biopsies of the gingiva in response to oral inflammation with adjustment for cell-type heterogeneities.

## Results

### Pre-processing pipeline

786,547 probes passed the quality control (QC) criteria and were analyzed in ex vivo gingival tissue biopsies from 60 periodontitis patients obtained from an inflamed and an uninflamed site each. Additionally, DNA methylation patterns of cultured gingival epithelial cells (GECs) and human gingival fibroblasts (HGFs), collected from 5 and 4 additional donors, respectively, and of immortalized cell lines of gingival epithelial (OKG4) and fibroblast (ihGF) cells, were analyzed.

### Suitability test of the EpiDish algorithm for the detection of gingival epithelial cells and gingival fibroblasts for cell type deconvolution

**Fig. 1:**
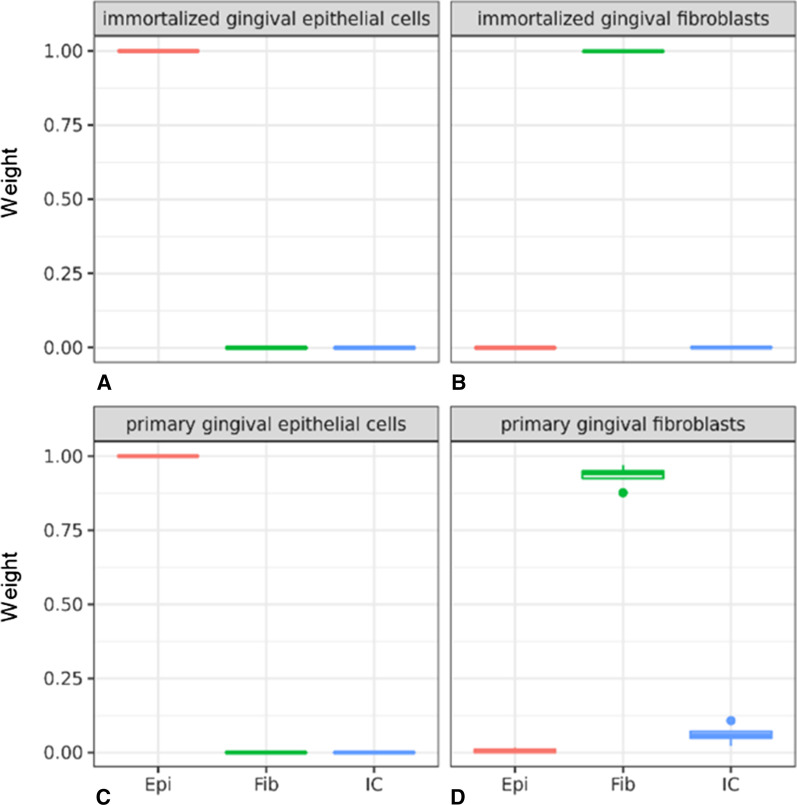
EpiDish results for gingival cell cultures. Cell type estimations in the cultured gingival cells inferred by EpiDish as average weight proportions of the major cell types epithelial cells (Epi), fibroblasts (Fib,) and immune cells (IC) for immortalized gingival epithelial cells (**A**) (*n* = 1), immortalized gingival fibroblasts (**B**) (*n* = 1), primary gingival epithelial cells (**C**) (*n* = 5), and primary gingival fibroblasts (**D**) (*n* = 4)

The cell type deconvolution algorithm EpiDish represents an established method to adjust for cell type heterogeneity in EWAS samples. It uses reference methylomes from various cell types. To test how the implemented reference methylomes of immune cells, epithelial cells, and fibroblasts matched the methylome of cells from gingival tissue, we analyzed the DNA from GECs and HGFs from cell culture on the EPIC BeadChip and performed cell type deconvolution with EpiDish. The primary GECs and both immortalized cell lines showed 100% concordance with the reference datasets (Fig. 1, Additional file 1). Primary gingival fibroblasts showed some deviations from the expected patterns, with 3 of the 4 samples showing an estimated fibroblast fraction of > 94%. One sample implied only 88% fibroblasts, with an infiltration of 11% immune cells and 2% epithelial cells.

### Estimation of the immune cell fraction in inflamed and uninflamed gingiva.

Substantial infiltration of immune cells into gingival tissues is disease-immanent in periodontitis. To account for differences in cell type composition, we applied the EpiDish algorithm to our 120 samples of paired inflamed an uninflamed ex vivo biopsies from 60 individuals. In the inflamed samples, half of the cell fraction of inflamed gingival tissues consisted of immune cells (mean 0.52, standard deviation (SD) 0.18). Some of the inflamed biopsies showed estimated immune cell fractions as high as 0.91 (Additional file 1). Uninflamed biopsies showed immune cell infiltration that was significantly lower compared to inflamed tissue (mean 0.28, SD 0.15; *p* < 10^–5^, Wilcoxon Test). In both inflamed and uninflamed samples, the amount of estimated immune cells was inversely correlated to the amount of epithelial cells in the samples (Pearson’s correlation coefficient = − 0.93). Estimations for epithelial cells accordingly were higher in uninflamed samples (mean 0.52, SD 0.15) than in inflamed samples (mean 0.32, SD 0.18). The degree of variation of immune cell infiltration was comparable between uninflamed and inflamed tissues, as was the degree of variation of the amount of epithelial cells. For 6 individuals, we observed higher immune cell fractions in clinically uninflamed samples compared to inflamed samples. The variation of the estimated amount of fibroblast cells in inflamed and uninflamed biopsies was lower and comparable between inflamed and uninflamed biopsies, with an estimated fibroblast fraction of 0.20 (SD 0.07) in uninflamed samples and 0.17 (SD 0.08) in inflamed samples.

**Fig. 2:**
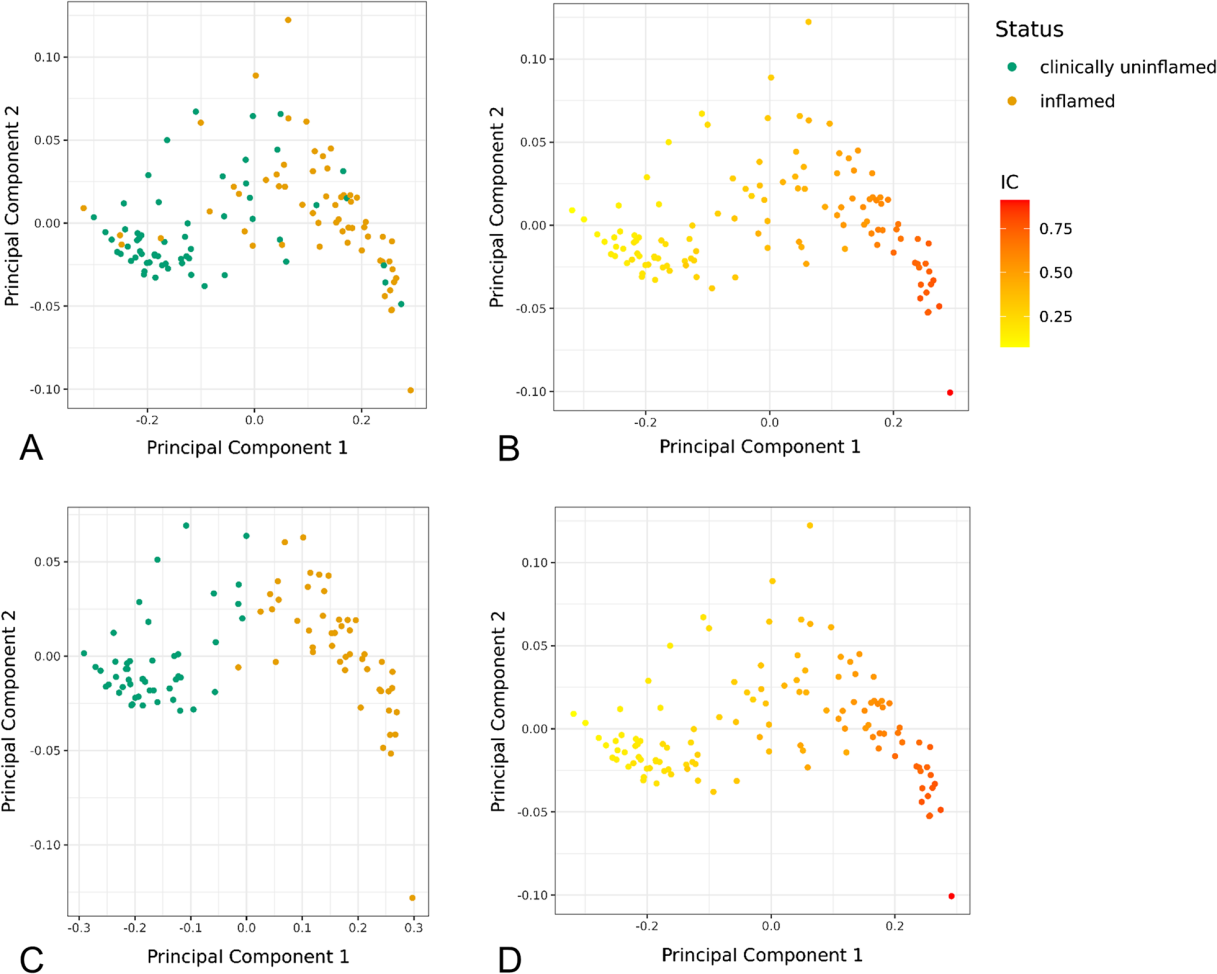
Multidimensional Scaling (MDS) Plots for inflamed and clinically uninflamed samples. **A** MDS Plot of the 120 samples from the 60 patients initially analyzed for cell-type heterogeneity using EpiDish, colored by diagnosed inflammation status. **B** MDS Plot of the 120 samples from the 60 patients initially analyzed for cell-type heterogeneity using EpiDish, colored according to the amount of infiltrated immune cells. **C** MDS Plot of the EpiDish-filtered 96 samples used for the EWAS, colored by diagnosed inflammation status. **D** MDS Plot of the EpiDish-filtered 96 samples used for the EWAS, colored according to the amount of infiltrated immune cells. In the initial sample panel, clinically uninflamed biopsies with high estimations of infiltrated immune cells clustered together with tissue diagnosed as inflamed (**A**, **B**). When including only samples diagnosed as “uninflamed” and “inflamed” that contained ≤ 35% and ≥ 40% immune cells, respectively, inflammation status (“uninflamed” and “inflamed”) clustered distinctly, with immune cell fractions as estimated by EpiDish reflecting this classification (**C**, **D**)

We furthermore performed a multidimensional scaling analysis to identify the effects of potential confounders on the methylome of gingival biopsies. This analysis revealed a separation according to the amount of immune cells as inferred by EpiDish, leading to the location of some biopsies that were clinically diagnosed as uninflamed in the cluster of inflamed biopsies (Fig. 2A, B). No clustering patterns were associated with the site of biopsy extraction (Additional file 2).

**Fig. 3:**
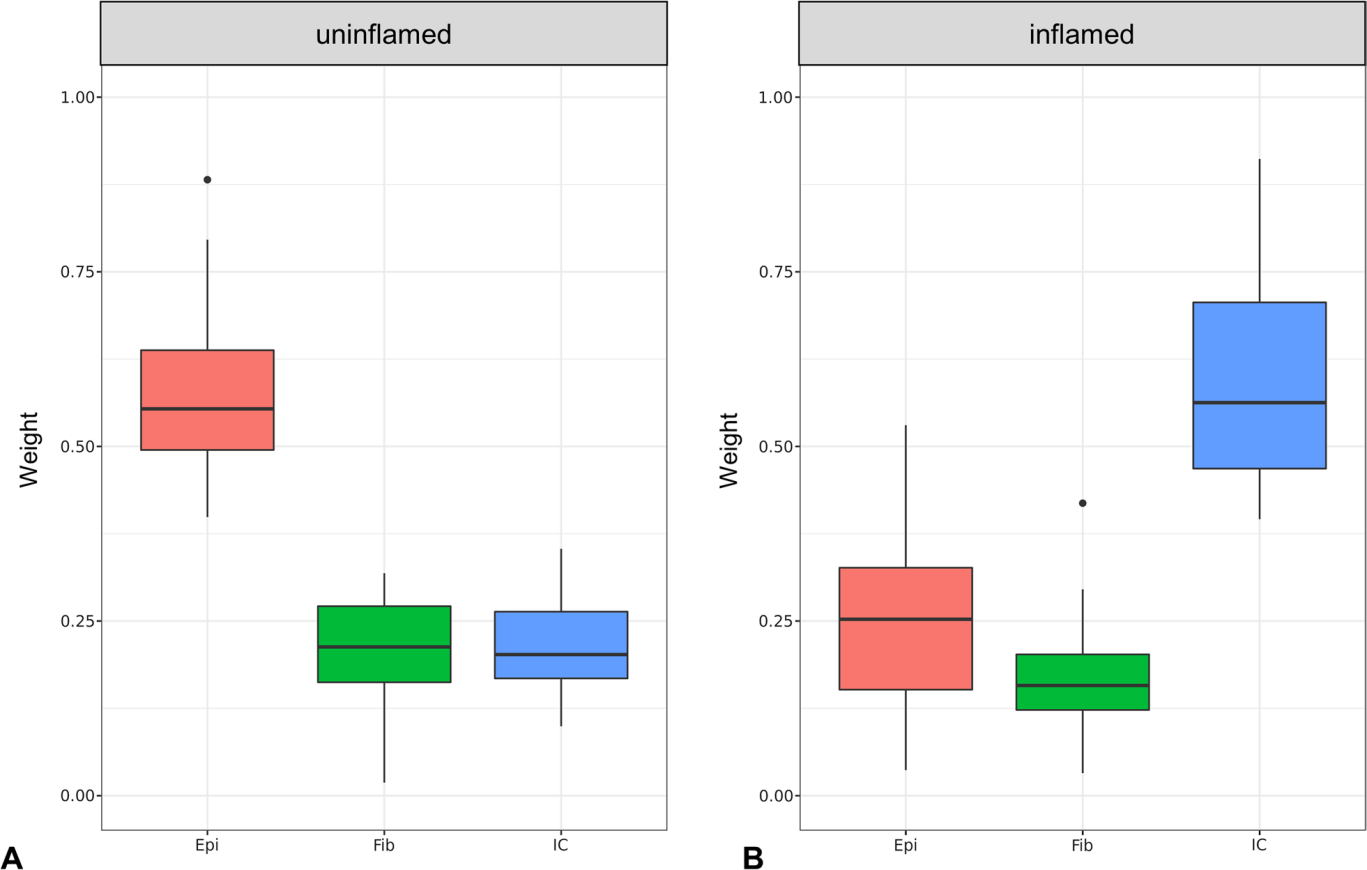
EpiDish results for the 96 samples selected for EWAS analysis. Cell type estimations in the gingiva inferred by EpiDish as average weight proportions of the major cell types epithelial cells (Epi), fibroblasts (Fib), and immune cells (IC) for uninflamed (**A**) and inflamed samples (**B**) (*n* = 48 each)

Based on the estimations of the cell type deconvolution, we excluded biopsies that were clinically diagnosed as uninflamed but appeared to be infiltrated by high amounts of immune cells, and those that were clinically diagnosed as inflamed but the estimated immune cell fraction was very low. Exclusion of these biopsies reduced confounding effects from putative misclassification or other factors. We set threshold criteria for sample inclusion as follows: samples diagnosed as “uninflamed” and “inflamed” contained ≤ 35% and ≥ 40% immune cells, respectively. In total, 48 samples from clinically uninflamed sites and 48 samples from inflamed sites complied with this criterion and were selected for subsequent association analyses. These 96 samples originated from 57 individuals, 39 of whom donated an uninflamed and an inflamed biopsy each. Thereafter, the mean estimation of immune cells was 0.22 (SD 0.06) for uninflamed and 0.58 (SD 0.13) for inflamed biopsies in the remaining 96 samples (Table 1, Fig. 3).

**Table 1:** Basic characteristics of the EWAS study population

	Uninflamed samples (*n* = 48)	Inflamed samples (*n* = 48)
Epithelial cells (mean)	0.57 ± 0.10	0.25 ± 0.12
Fibroblasts (mean)	0.21 ± 0.07	0.17 ± 0.07
Immune cells (mean)	0.22 ± 0.06	0.58 ± 0.13
Males, n (%)	28 (58.3)	30 (62.5)
Age, years (mean)	46.7 ± 9.7	45.7 ± 10.0
Smokers, n (%)	22 (45.8)	19 (39.6)

After removing individuals with immune cell fractions that contradicted their clinical classification from the analysis, samples clustered according to their clinical classification into inflamed and uninflamed tissue **(**Fig. 2C, D**).**

### DNA methylation differences of uninflamed and inflamed gingival biopsies

**Fig. 4:**
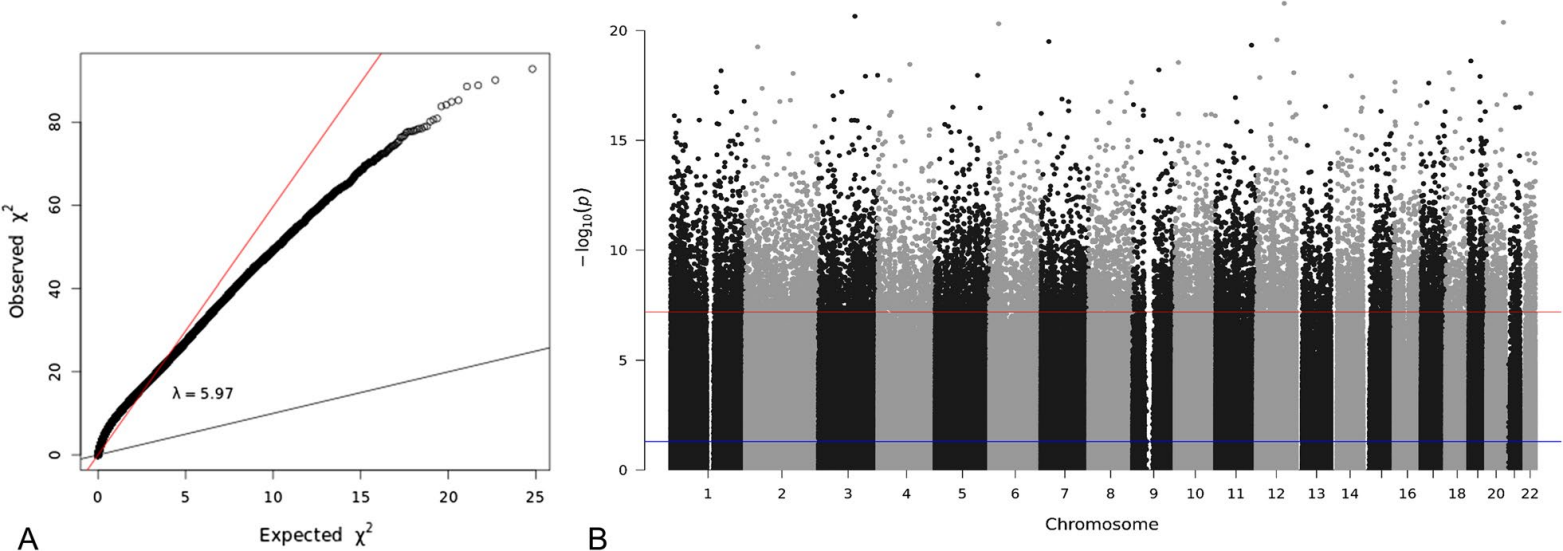
Manhattan and quantile–quantile plot for epigenome-wide associations with inflammation in the gingiva. **A** The quantile–quantile plot showed evidence for inflation of association signals (*λ* = 5.97). **B** Manhattan plot showing −log10 transformed *p* values plotted against the genomic location of the probes. The horizontal lines indicate the genome-wide significance threshold (*p* < 10^–7^)

**Fig. 5:**
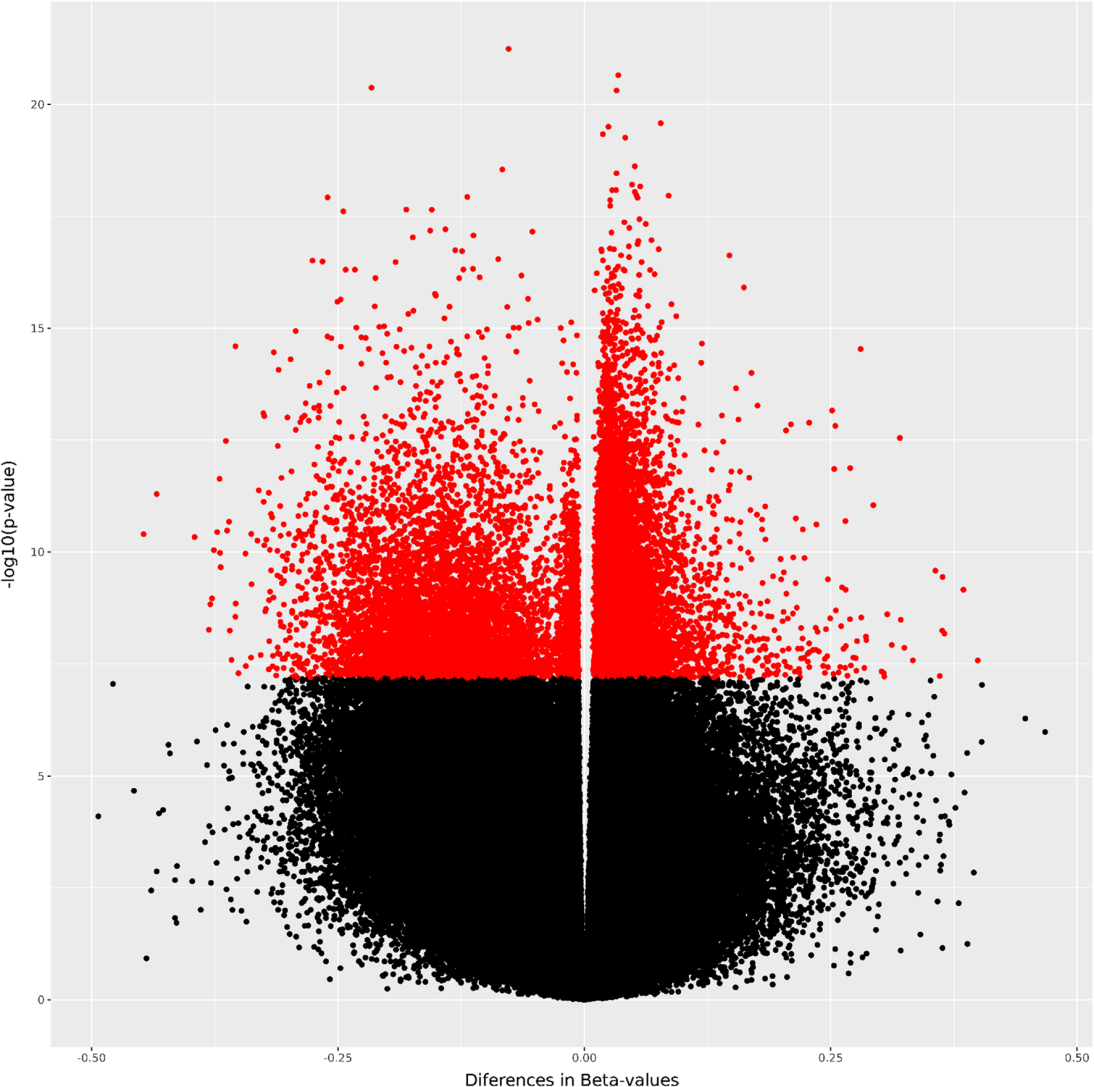
Volcano plot showing methylation differences of clinically uninflamed compared to inflamed samples against −log10 of *p* values. Depicted in red are the DMPs in periodontal inflammation significant after adjustment for multiple testing. Note that effect sizes were derived from *M*-values using our intercept-method [16], taking confounding factors, especially cell type heterogeneity, into account

Next, the selected samples were investigated for significant changes in methylation patterns between clinically uninflamed and inflamed samples in the 786,547 CpG sites that passed QC, with adjustment for batch effects and differences in immune cell content. Furthermore, as smoking is one of the major risk factors for periodontitis, 1501 DMPs associated with smoking in oral epithelial cells [14] were removed from the analysis. The quantile–quantile plot revealed global inflation of the test statistics compared to the expected distribution, with an inflation factor of *λ* = 5.97 (Fig. 4A). After correction for multiple testing, we found 15,507 DMPs with *q* < 0.05 (Figs. 4B, 5).

Intra-individual variation in DNA methylation is a strong confounder in epigenetic studies, which can be precluded by analyzing clinically uninflamed and inflamed biopsies of the same patients. Likewise, we observed that of the top 10 associated DMPs (best *q* = 4.5 × 10^–16^ at cg19478962, locating to the noncoding RNA gene *LOC643339*), only three showed nominally significant associations in a sub-analysis of the 39 individuals for whom an uninflamed and inflamed sample each was available for the EWAS. Considering this and the genomic inflation factor of 5.97, a filtering strategy was applied on the associated DMPs to minimize the possibilities of false positive associations due to inter-individual variation.

### DMPs in the full EWAS panel and a subset of paired inflamed and clinically uninflamed samples from the same individuals

**Fig. 6:**
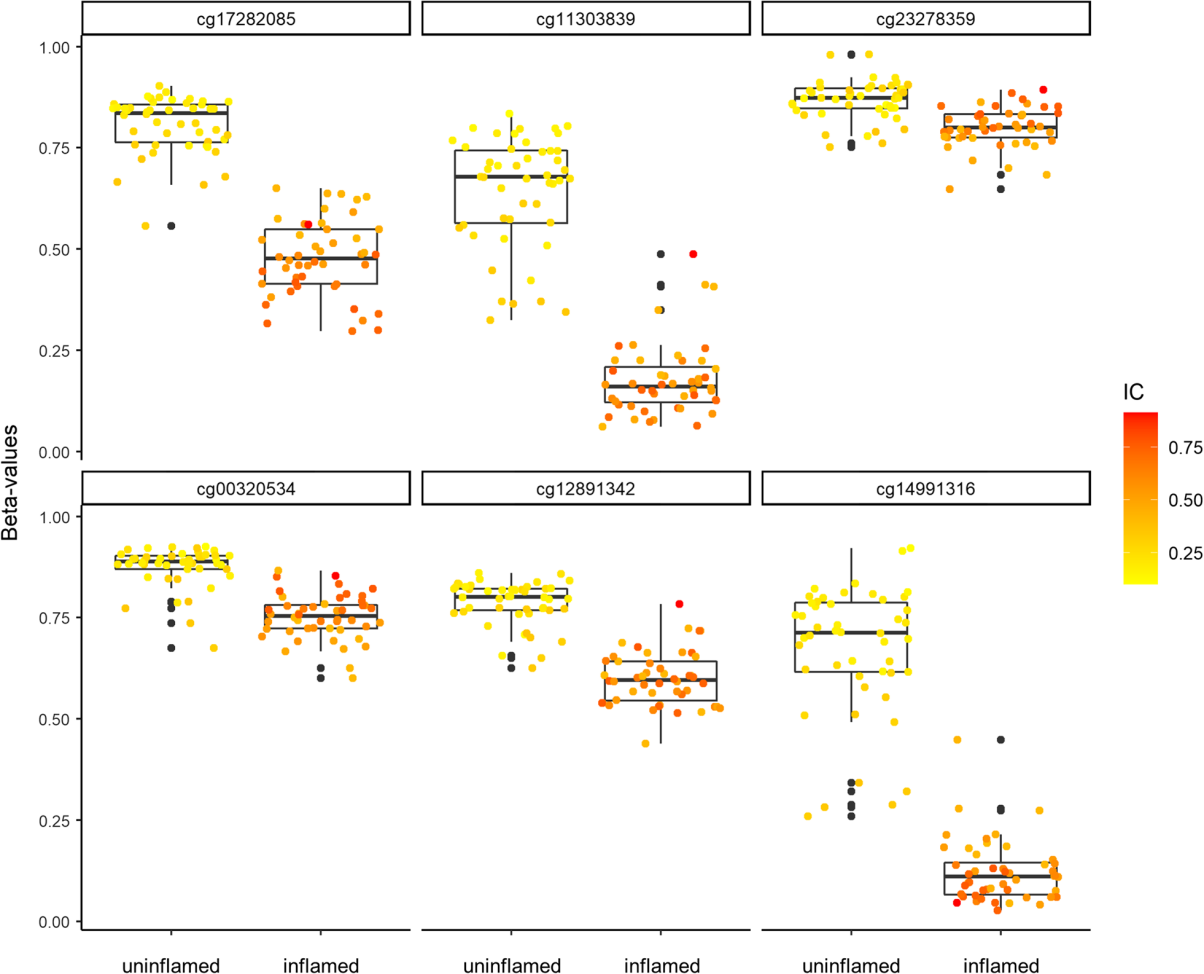
Methylation at top associated loci. Raw beta values of uninflamed and inflamed EWAS samples (full set, *n* = 48 each) at cg17282085 (*ROBO2*), cg11303839 (*CCL26*), cg23278359 (*PTP4A3*), cg00320534 (*DNAJC1*), cg12891342 (*LPXN*), and cg14991316 (*BPI*). IC = immune cell fraction in samples

We tested all 15,507 DMPs that were significant in the full sample panel of 57 EpiDish-filtered individuals (48 uninflamed and 48 inflamed samples, “full analysis”), which gave the largest power and thus reduced the potential of false negative findings, in the subset of paired samples (both inflamed and uninflamed samples from 39 individuals, “paired analysis”), to further reduce the potential of false positive findings. In this sub-analysis, 2347 DMPs showed significant associations with periodontal inflammation in the gingiva after Bonferroni-correction for 15,507 tests (Additional file 3). The 20 most significant DMPs showed *p* values < 10^–15^ and adjusted ∆*β* ≥ 0.05 in the full analysis (Table 2). The most significant DMP (cg23278359, Fig. 6) mapped to the gene *PTP4A3* (Protein Tyrosine Phosphatase 4A3) with *p* = 2.2 × 10^–18^, *q* = 1.8 × 10^–12^, and ∆*β* = − 0.18. The second most significant DMP mapped to the gene *ROBO2* (Roundabout Guidance Receptor 2; cg17282085; *q* = 4.9 × 10^–12^, ∆*β* = − 0.14), which is a suggestive risk gene for periodontitis (age 20–60 years; rs264537-C, *p* = 3 × 10^–6^, odds ratio = 1.35 [95% confidence interval 1.19–1.54]) [17]. Of the 2347 DMPs significant in the full and the paired analysis, 63 showed adjusted effect sizes > 0.3, with the 20 most pronounced effect sizes ≥ 0.34 (Table 3). The DMP with the highest effect size mapped to the gene *CCL26* (C–C Motif Chemokine Ligand 26; cg11303839, *q* = 4.0 × 10^–6^, ∆*β* = − 0.43). 3 of the top 20 associated DMPs and 2 of the 20 DMPs with the highest effect sizes located < 2 kb to other DMPs with *q* < 0.05 in the full analysis.

**Table 2:** Most significant DMPs in full analysis that were also significant in the paired sub-analysis

Probe ID	Chr:Position (hg19)	Distance to nearest significant DMP (bp)	Gene Symbol	Median TPM*	*p* (full analysis)	*q* (full analysis)	*p*_adj_ (paired analysis)	∆*β* (full analysis)	Raw beta values (mean)			
									Uninflamed (*n* = 48)	Inflamed (*n* = 48)	pGECs (*n* = 5)	Blood (*n* = 69)**
cg23278359	chr8:142,439,128	131	*PTP4A3*	78	2.2 × 10^–18^	1.8 × 10^–12^	0.01	− 0.18	0.87	0.8	0.8	0.93
cg17282085	chr3:77,684,050	455,069	*ROBO2*	239	6.2 × 10^–18^	4.9 × 10^–12^	8.8 × 10^–3^	− 0.14	0.81	0.48	0.55	0.57
cg06819431	chr1:153,605,367	25,710	*CHTOP*	701	6.6 × 10^–18^	5.2 × 10^–12^	1.3 × 10^–4^	− 0.16	0.89	0.78	0.89	0.93
cg25428009	chr8:126,304,221	194,357	*NSMCE2*	263	7.0 × 10^–18^	5.5 × 10^–12^	1.0 × 10^–4^	− 0.05	0.89	0.69	0.88	0.67
cg24469152	chr20:62,378,611	7815	*ZBTB46*	29	8.5 × 10^–18^	6.7 × 10^–12^	0.03	− 0.11	0.87	0.88	0.86	0.96
cg06405792	chr19:36,483,062	60,585	*SDHAF1*	153	1.8 × 10^–17^	1.4 × 10^–11^	7.1 × 10^–4^	− 0.13	0.88	0.81	0.89	0.92
cg15834202	chr5:58,865,918	38,967	*PDE4D*	79	3.1 × 10^–17^	2.4 × 10^–11^	4.4 × 10^–5^	− 0.28	0.77	0.45	0.65	0.61
cg27288968	chr21:34,106,765	116,334	*PAXBP1*	310	3.2 × 10^–17^	2.6 × 10^–11^	9.5 × 10^–4^	− 0.27	0.82	0.64	0.82	0.87
cg17835169	chr5:149,281,308	58,550	*PDE6A*	16	3.3 × 10^–17^	2.6 × 10^–11^	1.0 × 10^–3^	− 0.19	0.88	0.6	0.93	0.75
cg21500126	chr8:119,372,894	186,008	*SAMD12*	214	4.9 × 10^–17^	3.9 × 10^–11^	6.7 × 10^–5^	− 0.24	0.79	0.6	0.8	0.84
cg02967821	chr8:30,582,335	18,888	*GSR*	671	4.9 × 10^–17^	3.9 × 10^–11^	8.7 × 10^–5^	− 0.23	0.89	0.79	0.81	0.91
cg17423032	chr5:31,755,265	23,874	*PDZD2*	1696	1.9 × 10^–16^	1.5 × 10^–10^	3.6 × 10^–4^	− 0.15	0.89	0.77	0.91	0.93
cg19962349	chr5:43,534,107	69,246	*PAIP1*	1157	2.3 × 10^–16^	1.8 × 10^–10^	2.0 × 10^–5^	− 0.25	0.67	0.5	0.75	0.74
cg16084117	chr20:47,188,436	49,123	TRNA_Pseudo	NA	3.3 × 10^–16^	2.6 × 10^–10^	2.8 × 10^–3^	− 0.14	0.91	0.78	0.92	NA
cg01379678	chr18:32,397,156	6921	*DTNA*	11	4.1 × 10^–16^	3.2 × 10^–10^	1.2 × 10^–5^	− 0.17	0.85	0.72	0.88	0.93
cg11022756	chr4:170,540,250	6371	*CLCN3*	2771	6.0 × 10^–16^	4.8 × 10^–10^	6.9 × 10^–3^	− 0.14	0.83	0.75	0.85	0.92
cg00414647	chr19:40,781,453	57,065	*AKT2*	2076	7.7 × 10^–16^	6.0 × 10^–10^	0.01	− 0.06	0.9	0.76	0.92	0.82
cg27597264	chr6:8,086,870	232	*SCARNA27*	NA	9.1 × 10^–16^	7.1 × 10^–10^	1.5 × 10^–4^	− 0.20	0.85	0.7	0.87	0.89
cg27168410	chr19:41,629,018	1528	*CYP2F1*	41	9.3 × 10^–16^	7.4 × 10^–10^	6.2 × 10^–3^	− 0.21	0.88	0.71	0.89	0.9
cg16302962	chr8:131,124,760	187,033	*ASAP1*	623	9.7 × 10^–16^	7.7 × 10^–10^	0.02	− 0.23	0.85	0.62	0.92	0.84

20 most significant DMPs from the full analysis that also showed a significant association in the paired sub-analysis. *p* Values from paired analysis are Bonferroni-corrected for 15,507 tests. ∆*β*-values are adjusted according to our intercept method [16]. *mRNA-sequencing in healthy keratinized oral mucosa with 16mio reads/sample [12]. **downloaded from GSE123914 [54]. DMP = differentially methylated position, bp = basepairs, TPM = transcripts per million, pGECs = primary gingival epithelial cells

**Table 3.** 20 DMPs with highest effect sizes

Probe ID	Chr:Position (hg19)	Distance to nearest significant DMP (bp)	Gene Symbol	Median TPM*	*p* (full analysis)	*q* (full analysis)	*p*_adj_ (paired analysis)	∆*β* (full analysis)	Raw beta values (mean)			
									Uninflamed (*n* = 48)	Inflamed (*n* = 48)	pGECs (*n* = 5)	Blood (*n* = 69)**
cg11303839	chr7:75,405,967	84,519	*CCL26*	10	5.1 × 10^–12^	4.0 × 10^–6^	4.0 × 10^–4^	− 0.43	0.64	0.18	0.61	0.31
cg16307144	chr19:38,704,933	14,590	*DPF1*	6	4.7 × 10^–11^	3.6 × 10^–5^	0.012	− 0.40	0.68	0.31	0.57	0.66
cg08670006	chr14:56,738,739	154,092	*PELI2*	66	3.6 × 10^–11^	2.8 × 10^–5^	1.5 × 10^–3^	− 0.37	0.52	0.27	0.30	0.86
cg26189827	chr7:40,338,925	11,044	*SUGCT*	45	2.3 × 10^–12^	1.8 × 10^–6^	2.1 × 10^–3^	− 0.37	0.57	0.18	0.08	0.38
cg03606473	chr3:102,846,710	1,035,074	gene desert	NA	1.1 × 10^–10^	8.2 × 10^–5^	7.4 × 10^–5^	− 0.37	0.57	0.27	0.35	0.76
cg04025284	chr14:60,636,610	336,907	*DHRS7*	460	2.2 × 10^–10^	1.7 × 10^–4^	3.8 × 10^–3^	− 0.37	0.79	0.68	0.80	0.90
cg25249713	chr4:177,045,356	28,224	*WDR17*	9	2.2 × 10^–10^	1.7 × 10^–4^	0.016	− 0.37	0.78	0.35	0.88	0.83
cg10659908	chr12:109,796,147	66,574	*LINC01486*	NA	3.3 × 10^–13^	2.6 × 10^–7^	6.9 × 10^–4^	− 0.36	0.70	0.30	0.85	0.54
cg07804660	chr11:266,869	22,085	gene desert	NA	3.3 × 10^–11^	2.6 × 10^–5^	1.8 × 10^–4^	− 0.36	0.72	0.40	0.80	0.79
cg24852319	chr6:113,278,378	266,486	gene desert	NA	2.1 × 10^–11^	1.7 × 10^–5^	1.2 × 10^–4^	− 0.36	0.71	0.36	0.55	0.83
cg27171156	chr2:71,698,651	4367	DYSF	92	5.6 × 10^–9^	4.4 × 10^–3^	1.8 × 10^–3^	− 0.36	0.61	0.31	0.11	0.79
cg27531927	chr13:98,598,462	29,598	gene desert	NA	2.5 × 10^–8^	0.020	3.0 × 10^–3^	− 0.36	0.61	0.27	0.23	0.74
cg09168806	chr8:97,056,373	523	gene desert	NA	2.8 × 10^–9^	2.2 × 10^–3^	2.7 × 10^–3^	− 0.35	0.61	0.30	0.24	0.75
cg01306824	chr17:77,834,118	18,952	gene desert	NA	2.5 × 10^–15^	2.0 × 10^–9^	0.010	− 0.35	0.63	0.50	0.67	0.94
cg01658895	chr5:74,621,313	11,545	gene desert	NA	1.4 × 10^–9^	1.1 × 10^–3^	1.2 × 10^–5^	− 0.35	0.63	0.42	0.76	0.84
cg02061660	chr5:110,088,130	12,074	*SLC25A46*	747	5.1 × 10^–8^	0.039	5.8 × 10^–4^	− 0.35	0.68	0.40	0.70	0.84
cg09605880	chr1:32,397,195	7131	*PTP4A2*	1034	1.1 × 10^–10^	8.5 × 10^–5^	2.8 × 10^–3^	− 0.34	0.51	0.21	0.47	0.52
cg12859373	chr6:90,993,998	203,279	*BACH2*	135	2.3 × 10^–8^	0.017	7.0 × 10^–3^	− 0.34	0.54	0.10	0.24	0.14
cg14991316	chr20:36,932,377	8	*BPI*	0	4.0 × 10^–11^	3.1 × 10^–5^	1.0 × 10^–3^	− 0.34	0.67	0.12	0.92	0.06
cg19996026	chr10:69,455,804	262,127	*CTNNA3*	2	5.2 × 10^–10^	4.1 × 10^–4^	1.7 × 10^–3^	− 0.34	0.67	0.35	0.66	NA

DMPs with the highest effect sizes from the full analysis that also showed a significant association in the paired sub-analysis. *p* Values from paired analysis are Bonferroni-corrected for 15,507 tests. ∆*β*-values are adjusted according to our intercept method [16]. *mRNA-sequencing in healthy keratinized oral mucosa with 16mio reads/sample [12]. **downloaded from GSE123914 [54]. DMP = differentially methylated position, bp = basepairs, TPM = transcripts per million, pGECs = primary gingival epithelial cells

Subsequently, a more stringent filtering process for the 15,507 DMPs that were significant in the full analysis was applied to further reduce the number of putative random associations. These filtering criteria were: (1) ≥ 2 significant DMPs (*q* < 0.05 in the full analysis) had a maximum distance of 2 kb, (2) ≥ 1 DMP of such a cluster showed effect sizes > 0.1 in the full analysis, (3) ≥ 1 DMP of such a cluster showed *p*_adj_ < 0.05 in the paired analysis. This resulted in 441 DMPs in 193 clusters, with *p* values < 6.5 × 10^–8^ in the full analysis (Additional file 4). Of these, 22 DMPs showed *p*_adj_ < 10^–5^ in the paired analysis (Table 4).

**Table 4:** DMPs associated with *p*_adj_ < 10^–5^ in the paired sub-analysis after stringent filtering

Probe ID	Chr:Position (hg19)	Distance to nearest significant DMP (bp)	Sup-porting DMPs (n)	Gene Symbol	Median TPM*	*p* (full analysis)	*q* (full analysis)	*p*_adj_ (paired analysis)	∆*β* (full analysis)	Raw beta values (mean)			
										Uninflamed (*n* = 48)	Inflamed (*n* = 48)	pGECs (*n* = 5)	Blood* (*n* = 69)
cg00320534	chr10:22,216,647	201	1	*DNAJC1*	391	1.3 × 10^–15^	1.0 × 10^–9^	9.1 × 10^–6^	− 0.20	0.88	0.75	0.24	0.92
cg12891342	chr11:58,344,244	90	5	*LPXN*	64	4.3 × 10^–8^	3.4 × 10^–2^	1.4 × 10^–5^	− 0.20	0.79	0.6	0.83	0.88
cg07215395	chr5:115,148,221	1251	1	CDO1	12	1.3 × 10^–14^	9.9 × 10^–9^	2.9 × 10^–5^	− 0.20	0.83	0.62	0.80	0.90
cg11470748	chr15:92,378,414	102	1	*SLCO3A1*	482	2.1 × 10^–8^	1.6 × 10^–2^	4.1 × 10^–5^	− 0.18	0.64	0.55	0.67	0.90
cg07994786	chr16:48,389,355	409	1	*LONP2*	1010	3.5 × 10^–10^	2.7 × 10^–4^	4.7 × 10^–5^	− 0.15	0.86	0.78	0.88	0.95
cg02099194	chr13:43,149,689	34	1	*TNFSF11*	1	4.8 × 10^–12^	3.8 × 10^–6^	6.3 × 10^–5^	− 0.19	0.36	0.18	0.46	0.27
cg25157850	chr20:62,401,287	78	2	*ZBTB46*	29	8.4 × 10^–10^	6.5 × 10^–4^	6.5 × 10^–5^	− 0.23	0.77	0.62	0.68	0.91
cg08446539	chr12:25,541,565	201	1	*KRAS/LMNTD1*	730 / 0	9.1 × 10^–12^	7.1 × 10^–6^	6.5 × 10^–5^	− 0.24	0.79	0.5	0.81	0.75
cg06142324	chr11:124,805,538	425	1	*HEPACAM*	0	1.8 × 10^–11^	1.4 × 10^–5^	7.8 × 10^–5^	− 0.13	0.85	0.77	0.82	0.93
cg18038139	chr17:10,560,748	72	2	*MYH3*	24	1.3 × 10^–8^	9.8 × 10^–3^	8.0 × 10^–5^	− 0.20	0.7	0.55	0.75	0.90
cg19705197	chr10:6,264,090	606	1	*PFKFB3*	408	3.6 × 10^–12^	2.8 × 10^–6^	8.2 × 10^–5^	− 0.20	0.74	0.69	0.68	0.93
cg10254357	chr1:95,592,493	162	1	*TMEM56*	22	4.4 × 10^–12^	3.4 × 10^–6^	8.4 × 10^–5^	− 0.20	0.8	0.64	0.76	0.89
cg09083279	chr6:29,454,873	15	2	*MAS1L*	0	4.9 × 10^–12^	3.8 × 10^–6^	8.9 × 10^–5^	− 0.27	0.56	0.34	0.37	0.63

DMPs meeting the following filter criteria: *q* < 0.05, effect sizes ≥ 0.1, distance to nearest significant (*q* < 0.05) DMP $\le$ 2 kb (= supporting DMP), *p*_adj_ < 10^–5^ in paired analysis (*n* = 39), ranked according to their p_adj_ in the paired analysis. *p* Values from paired analysis are Bonferroni-corrected for 15,507 tests. ∆*β*-values are adjusted according to our intercept method [16]. *mRNA-sequencing in healthy keratinized oral mucosa with 16mio reads/sample [12]. **downloaded from GSE123914 [54]. DMP = differentially methylated position, bp = basepairs, TPM = transcripts per million pGECs = primary gingival epithelial cells

The most significant DMP in the paired analysis (cg00320534, _adj_*p*_paired_ = 9.1 × 10^–6^, ∆*β* = − 0.2, 1 supporting DMP) located to the gene *DNAJC1* (DnaJ Heat Shock Protein Family Member C1). The second most significant DMP located to the gene *LPXN* (Leupaxin) and was supported by 5 additional DMPs that showed *q*-values < 0.05 in the full analysis (cg12891342, _adj_*p*_paired_ = 1.4 × 10^–5^, ∆*β* = − 0.2). Subsequently, we ranked the 441 DMPs according to their effect size delta beta (in the full analysis). 7 DMPs showed effect sizes > 0.3 (Table 5). The DMP with the largest effect size from this filtering approach (cg14991316, _adj_*p*_paired_ = 1.0 × 10^–3^, ∆*β* = − 0.34, 3 supporting DMPs) mapped to the promoter region of *BPI* (Bactericidal Permeability Increasing Protein).

**Table 5:** DMPs with effect sizes ≥ 0.3 after stringent filtering

Probe ID	Chr:Position (hg19)	Distance to nearest significant DMP (bp)	Sup-porting DMPs (n)	Gene Symbol	Median TPM*	*p* (full analysis)	*q* (full analysis)	*p*_adj_ (paired analysis)	∆*β* (full analyis)	Raw beta values (mean)			
										Uninflamed (*n* = 48)	Inflamed (*n* = 48)	pGECs (*n* = 5)	Blood** (*n* = 69)
cg14991316	chr20:36,932,377	8	*3*	*BPI*	0	4.0 × 10^–11^	3.1 × 10^–5^	1.0 × 10^–3^	− 0.34	0.67	0.12	0.92	0.06
cg09514933	chr2:242,605,566	185	*3*	*ATG4B*	554	7.9 × 10^–14^	6.2 × 10^–8^	1.4 × 10^–3^	− 0.33	0.89	0.76	0.94	0.96
cg10539002	chr10:117,061,032	561	*1*	*ATRNL1*	22	9.5 × 10^–11^	7.4 × 10^–5^	1.3 × 10^–3^	− 0.31	0.53	0.28	0.42	0.67
cg11804639	chr8:97,056,896	523	*1*	*GAPDHP30*	NA	1.2 × 10^–10^	9.7 × 10^–5^	3.5 × 10^–3^	− 0.31	0.77	0.37	0.66	NA
cg15528852	chr7:41,137,006	40	*1*	*LINC01449*	NA	1.9 × 10^–8^	1.5 × 10^–2^	5.0 × 10^–4^	− 0.31	0.68	0.51	0.64	0.85
cg18450582	chr7:95,546,539	31	*1*	*DYNC1I1*	15	2.4 × 10^–10^	1.8 × 10^–4^	9.6 × 10^–4^	− 0.30	0.80	0.64	0.82	0.94
cg27130240	chr16:88,943,533	356	*3*	*CBFA2T3*	72	9.3 × 10^–9^	7.2 × 10^–3^	7.5 × 10^–2^	− 0.30	0.72	0.74	0.64	0.99

DMPs meeting the following filter criteria: *q* < 0.05, distance to nearest significant (*q* < 0.05) DMPs $\le$ 2 kb (= supporting DMPs), effect size ≥ 0.3, at least one DMP in cluster with p_adj_ paired < 0.05. *p* Values from paired analysis are Bonferroni-corrected for 15,507 tests. ∆*β*-values are adjusted according to our intercept method [16]. *mRNA-sequencing in healthy keratinized oral mucosa with 16mio reads/sample [12]. **downloaded from GSE123914 [54]. DMP = differentially methylated position, bp = basepairs, TPM = transcripts per million, pGECs = primary gingival epithelial cells

### Identification of differentially methylated regions

**Fig. 7:**
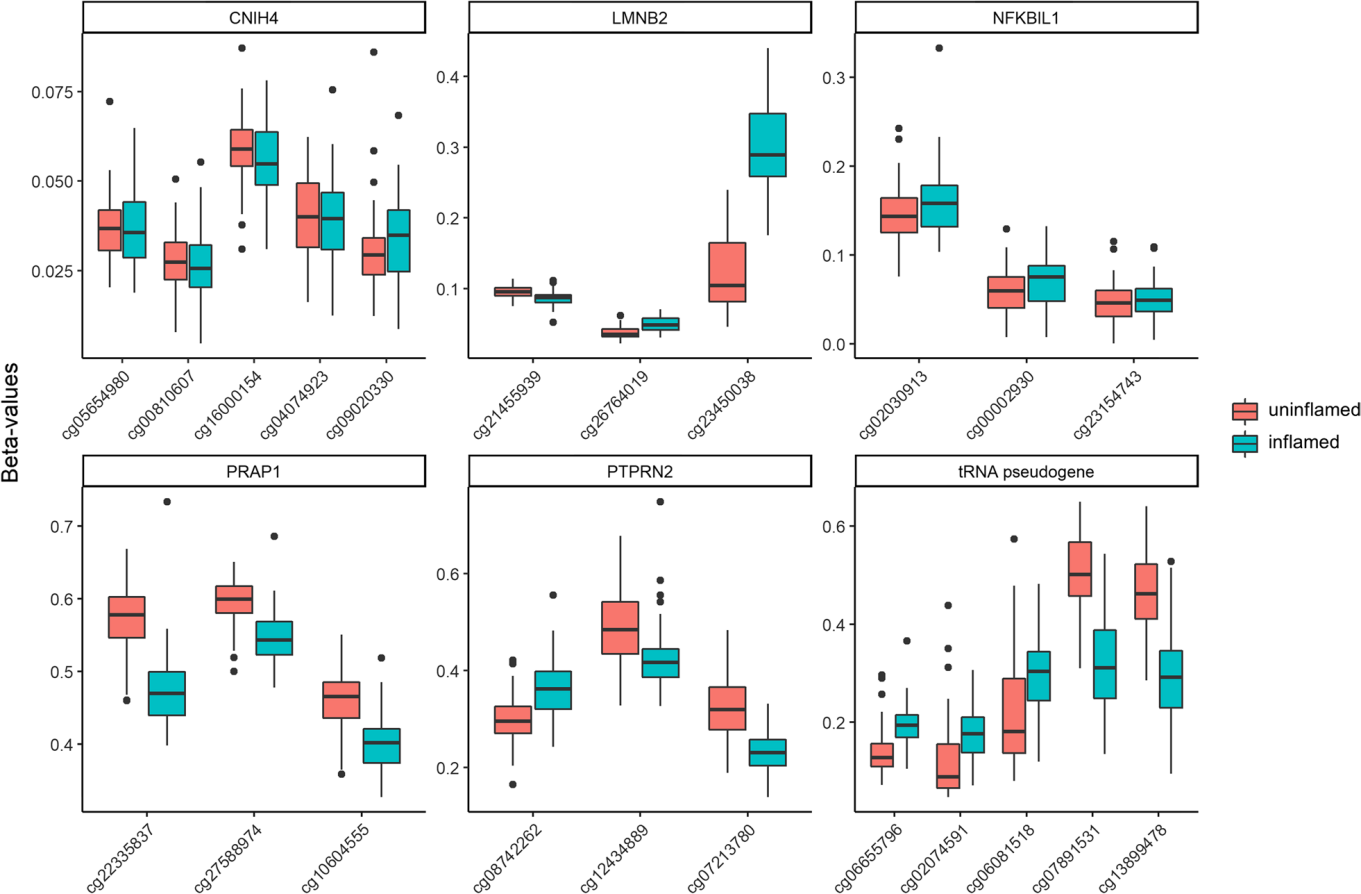
Methylation at significant differentially methylated regions. Raw beta values of uninflamed and inflamed EWAS samples (full set, *n* = 48 each) at the genetic regions of *CNIH4*, *LMNB2*, *NFKBIL1*, *PRAP1*, *PRPTN2*, and the tRNA pseudogene on chromosome 16

To identify differentially methylated regions (DMR), we applied the Bump Hunter algorithm [18] to our full dataset of 48 inflamed and 48 uninflamed samples and to the subset of paired samples. In the full dataset, 6 DMR showed a family-wise error rate (FWER) < 0.05, whereas in the paired sub-analysis, 11 DMR showed a FWER < 0.05 (Table 6). 6 DMR were significantly associated in both approaches, with a 151 base pair (bp) region at the transcription start site of *CNIH4* (Cornichon Family AMPA Receptor Auxiliary Protein 4) showing the strongest associations (Fig. 7).

**Table 6:** Summary of significant differentially methylated regions

Chr:position (start–end) (hg19)	Gene symbol	Median TPM*	Regulatory features**	Paired analysis		Full analysis	
				*p*	FWER	*p*	FWER
chr1:224,544,384–224,544,535	*CNIH4*	723	TSS; DHS	< 10^–6^	< 0.01	< 10^–6^	< 0.01
chr16:3,220,475–3,221,355	tRNA pseudogene	NA	R, E, TSS***	< 10^–6^	< 0.01	4.7 × 10^–4^	0.04
chr1:23,857,884–23,858,301	*E2F2*	154	TSS; DHS	9.8 × 10^–5^	0.01	n.s	n.s
chr11:8,986,449–8,986,674	*TMEM9B*	1020	TSS; DHS	9.8 × 10^–5^	0.01	n.s	n.s
chr6:31,515,391–31,515,404	*NFKBIL1*	128	TSS; DHS	9.8 × 10^–5^	0.01	1.2 × 10^–4^	0.01
chr7:158,342,283–15,8342,600	*PTPRN2*	39	R, T, WE***; DHS	9.8 × 10^–5^	0.01	5.9 × 10^–4^	0.04
chr10:135,164,246–135,164,482	*PRAP1*	0	T	9.8 × 10^–5^	0.01	5.9 × 10^–4^	0.04
chr19:2,462,441–2,462,961	*LMNB2*	1103	R, E, TSS***	2.0 × 10^–4^	0.02	5.9 × 10^–4^	0.04
chr11:64,360,872–64,360,997	*SLC22A12*	0	R	2.9 × 10^–4^	0.03	n.s	n.s
chr11:2,919,808–2,920,209	*SLC22A18* (+ -AS)	53 (5)	TSS, E, WE***; DHS	3.9 × 10^–4^	0.04	n.s	n.s
chr1:161,087,882–161,087,906	*NIT1*	238	TSS; DHS	3.9 × 10^–4^	0.04	n.s	n.s

^*^mRNA-sequencing in healthy keratinized oral mucosa with 16mio reads/sample [12]. **Based on ChIP-seq data from the ENCODE Consortium. ***depending on cell type. DNase hypersensitivity sites (DHS) with cluster score 1000 are listed. TPM = transcripts per million, FWER = family wise error rate, TSS = Predicted promoter region including transcription start site, E = Predicted enhancer, WE = Predicted weak enhancer or open chromatin cis regulatory element, T = Predicted transcribed region, R = Predicted repressed or low activity region, N.s. = not significant (*p* and FWER > 0.05, respectively)

### Gene ontology and gene set enrichment analysis

To determine whether the observed differential methylation is enriched in specific gene ontology (GO) terms or Kyoto Encyclopedia of Genes and Genomes (KEGG) pathways, we performed an enrichment analysis for the 2347 DMPs that were significantly associated in the full and the paired analysis. No GO term or KEGG pathway showed significant overrepresentation after adjustment for multiple testing (Table 7, Additional file 5). The most significant term (“inactivation of X chromosome by genetic imprinting”, *p*_unadj_ = 4.3 × 10^–5^) includes only three genes, *PCGF3* (Polycomb Group Ring Finger 3), *PCGF5* (Polycomb Group Ring Finger 5), and *PCGF6* (Polycomb Group Ring Finger 6), which all show significant DMPs in the EWAS and expression in healthy keratinized oral mucosa (transcripts per million (TPM) > 100), and are involved in chromatin modeling.

**Table 7:** Gene Ontology enrichment analysis

Sub-ontology	Gene ontology term	*N* genes in GO term	*N* differentially methylated genes (%)*	*p*_unadj_
BP	inactivation of *X* chromosome by genetic imprinting	3	3 (100.0%)	4.3 × 10^–5^
BP	synaptic vesicle cycle	191	18 (9.4%)	9.0 × 10^–5^
CC	hippocampal mossy fiber to CA3 synapse	33	7 (21.2%)	9.7 × 10^–5^
BP	vesicle-mediated transport in synapse	211	18 (8.5%)	3.0 × 10^–4^
MF	small conductance calcium-activated potassium channel activity	4	3 (75.0%)	3.1 × 10^–4^
BP	synaptic vesicle fusion to presynaptic active zone membrane	22	5 (22.7%)	3.5 × 10^–4^
CC	neuron projection	1298	64.5 (5.0%)	3.6 × 10^–4^
BP	vesicle fusion to plasma membrane	23	5 (21.7%)	4.0 × 10^–4^
BP	chemical synaptic transmission	699	40.5 (5.8%)	5.1 × 10^–4^
BP	anterograde trans-synaptic signaling	699	40.5 (5.8%)	5.1 × 10^–4^
BP	metanephric renal vesicle formation	4	3 (75.0%)	5.2 × 10^–4^
BP	neurotransmitter secretion	169	15 (8.9%)	5.8 × 10^–4^
BP	signal release from synapse	169	15 (8.9%)	5.8 × 10^–4^
BP	trans-synaptic signaling	707	40.5 (5.7%)	6.5 × 10^–4^
CC	synaptic vesicle	190	15 (7.9%)	6.7 × 10^–4^
MF	calcium-activated potassium channel activity	15	4 (26.7%)	8.1 × 10^–4^
MF	glutamate receptor activity	27	6 (22.2%)	9.7 × 10^–4^
BP	synaptic signaling	724	40.5 (5.6%)	9.9 × 10^–4^

Most significant gene ontology (GO) terms (*p* < 0.001) for genes corresponding to the 2347 DMPs significant in the full and paired sub-analysis. None of the pathways was significant after correction for multiple testing (FDR). *numbers are adjusted for probe-number bias and annotation to multiple genes by using a fractional count [19]. BP = biological process; MF = molecular function; CC = cellular component

Furthermore, when considering all GO pathways with nominal *p* values < 0.001, which might indicate a trend, there was an overrepresentation of GO terms corresponding to nervous system-related terms. As an example, we investigated the associations of the second most significant GO term, “synaptic vesicle cycle” (biological process, *p* = 9.0 × 10^–5^), more in detail, because this term included more genes compared to the most enriched GO term. 25 genes belonging to this GO term showed DMPs in the full and paired analysis, 10 of which with a *q* < 0.0001 in the full set (Table 8). From the top 10 differentially methylated genes in this GO term, STRING [20] indicated an interaction of 4 genes, *SNAP23* (Synaptosome Associated Protein 23), *SYT9* (Synaptotagmin 9), *STX1B* (Syntaxin 1B), and *SYT2* (Synaptotagmin 2) with the periodontitis-associated genes *VAMP3* [21, 22] and *VAMP8* (Vesicle Associated Membrane Protein 8; Additional file 6**)** [23]. All of these genes show relatively little to no expression in the healthy keratinized oral mucosa, determined by RNA-Sequencing of 39 samples [12], with exception of *CLCN3* and *SNAP23* (TPM = 2771 and 615, respectively). Notably, the location of the DMPs within these genes are predicted promotor flanking and transcribed regions as indicated by ENCODE [24], whereas the other, less expressed genes have significant DMPs within regions mapping to sites mainly predicted as repressed or low activity regions. Only 4 KEGG pathways showed nominal significance (*p* < 0.05), among which was “SNARE interactions in vesicular transport” with *p* = 0.036 (3rd most significant pathway, Additional file 5). Notably, the functionally related pathway “Mucin type O-glycan biosynthesis” was the 8^th^ most significant pathway out of 343 with *p* = 0.09.

**Table 8:** Differentially methylated genes within the gene ontology term “synaptic vesicle cycle” (biological process)

Gene	Full name	TPM*	Probe ID	Chr:Pos (hg19)	Regulatory features**	∆*β* (full analysis)	*p* (full analysis)	*q* (full analysis)	*p*_adj_ paired **
*CLCN3*	Chloride Voltage-Gated Channel 3	2771	cg11022756	chr4:170,540,250	PF	− 0.14	6.0 × 10^–16^	4.8 × 10^–10^	6.9 × 10^–3^
*RIMS1*	Regulating Synaptic Membrane Exocytosis 1	0	cg21984541	chr6:72,795,650	R	− 0.27	1.0 × 10^–13^	7.9 × 10^–8^	3.1 × 10^–4^
*SNAP23*	Synaptosome Associated Protein 23	615	cg13922935	chr15:42,804,870	T, R***	− 0.20	1.7 × 10^–12^	1.3 × 10^–6^	1.0 × 10^–5^
*SYT9*	Synaptotagmin 9	78	cg21672572	chr11:7,369,177	R	− 0.33	4.2 × 10^–12^	3.3 × 10^–6^	4.7 × 10^–5^
*STX1B*	Syntaxin 1B	21	cg05787209	chr16:31,022,521	R, E	− 0.19	6.9 × 10^–12^	5.4 × 10^–6^	0.03
*SYN3*	Synapsin III	1	cg16894211	chr22:33,403,542	R	− 0.24	1.6 × 10^–11^	1.3 × 10^–5^	1.6 × 10^–3^
*SYT2*	Synaptotagmin 2	1	cg03532262	chr1:202,587,916	R, WE	− 0.17	2.7 × 10^–11^	2.1 × 10^–5^	4.5 × 10^–4^
*SLC17A8*	Solute Carrier Family 17 Member 8	0	cg03817774	chr12:100,772,630	R	− 0.28	3.4 × 10^–11^	2.7 × 10^–5^	2.7 × 10^–3^
*SYNDIG1*	Synapse Differentiation Inducing 1	5	cg04332507	chr20:24,475,147	R, T	− 0.15	5.8 × 10^–11^	4.6 × 10^–5^	0.05
*RIMS2*	Regulating Synaptic Membrane Exocytosis 2	4	cg23568361	chr8:104,862,936	R	− 0.20	8.7 × 10^–11^	6.8 × 10^–5^	2.2 × 10^–3^
*CADPS*	Calcium Dependent Secretion Activator	12	cg17149019	chr3:62,432,102	R	− 0.14	2.7 × 10^–10^	2.1 × 10^–4^	3.4 × 10^–3^
*UNC13C*	Unc-13 Homolog C	6	cg15450958	chr15:54,812,261	R, T, E	− 0.22	1.2 × 10^–9^	9.7 × 10^–4^	0.04
*CPLX1*	Complexin 1	6	cg16649791	chr4:816,968	R	− 0.25	1.5 × 10^–9^	1.2 × 10^–3^	4.5 × 10^–3^
*SYT9*	Synaptotagmin 9	78	cg07649045	chr11:7,260,764	R	− 0.13	1.6 × 10^–9^	1.3 × 10^–3^	1.3 × 10^–4^
*ERC2*	ELKS/RAB6-Interacting/CAST Family Member 2	62	cg24265969	chr3:55,654,481	E, R	− 0.32	2.1 × 10^–9^	1.6 × 10^–3^	0.05
*PLD1*	Phospholipase D1	1469	cg06085579	chr3:171,509,822	TSS, E, T, WE	− 0.17	3.1 × 10^–9^	2.4 × 10^–3^	1.3 × 10^–3^
*SYT7*	Synaptotagmin 7	103	cg05131646	chr11:61,324,780	R, WE	− 0.12	3.4 × 10^–9^	2.7 × 10^–3^	8.7 × 10^–3^
*SYT12*	Synaptotagmin 12	6	cg13851621	chr11:66,812,262	R, CTCF	− 0.19	3.6 × 10^–9^	2.8 × 10^–3^	7.7 × 10^–3^
*RAB3B*	RAB3B, Member RAS Oncogene Family	14	cg01223789	chr1:52,392,189	CTCF	− 0.23	3.8 × 10^–9^	3.0 × 10^–3^	4.5 × 10^–3^
*SLC17A8*	Solute Carrier Family 17 Member 8	0	cg17713193	chr12:100,803,749	R	− 0.22	4.6 × 10^–9^	3.6 × 10^–3^	0.05
*SYT1*	Synaptotagmin 12	12	cg16495154	chr12:79,802,105	CTCF, E	− 0.18	5.9 × 10^–9^	4.6 × 10^–3^	4.4 × 10^–3^
*SYNJ2*	Synaptojanin 2	251	cg19920417	chr6:158,468,344	R, T, WE	− 0.21	7.4 × 10^–9^	5.8 × 10^–3^	9.2 × 10^–3^
*BSN*	Bassoon Presynaptic Cytomatrix Protein	7	cg20796968	chr3:49,700,419	R	− 0.09	1.3 × 10^–8^	0.01	0.01
*CPLX2*	Complexin 2	5	cg14416206	chr5:175,247,405	R	− 0.18	1.3 × 10^–8^	0.01	2.9 × 10^–3^
*DDC*	Dopa Decarboxylase	0	cg08241694	chr7:50,633,896	R, PF	− 0.14	1.5 × 10^–8^	0.01	0.03
*RIMS1*	Regulating Synaptic Membrane Exocytosis 1	0	cg26298612	chr6:73,074,746	R	− 0.11	2.1 × 10^–8^	0.02	2.9 × 10^–3^
*DNM1L*	Dynamin 1 Like	867	cg08823831	chr12:32,871,622	T	− 0.16	2.1 × 10^–8^	0.02	7.8 × 10^–3^
*SYT7*	Synaptotagmin 7	103	cg02284433	chr11:61,280,813	R, WE	− 0.13	5.3 × 10^–8^	0.04	0.04
*PRKAR1B*	Protein Kinase CAMP-Dependent Type I Regulatory Subunit β	176	cg26669717	chr7:641,028	R, T, TSS	− 0.11	5.7 × 10^–8^	0.04	0.04
*CADPS*	Calcium Dependent Secretion Activator	12	cg03546671	chr3:62,789,196	R	− 0.15	5.8 × 10^–8^	0.04	6.3 × 10^–3^

Genes from the gene ontology term “synaptic vesicle cycle” with significant DMPs [*q* (full analysis) ≤ 0.05 and *p*_adj_ (paired) ≤ 0.05], sorted by *p* values (full analysis). ∆*β*-values are adjusted according to our intercept method [16]. *mRNA-sequencing in healthy keratinized oral mucosa with 16mio reads/sample [12]. **Based on Chip-Seq Data from the ENCODE project. **^*^in H1-hESC (H1 human embryonic stem cell line) only. TSS = Predicted promoter region including transcription start site, PF = Predicted promoter flanking region, E = Predicted enhancer, WE = Predicted weak enhancer or open chromatin cis regulatory element, CTCF = CTCF enriched element, T = Predicted transcribed region, R = Predicted repressed or low activity region

## Discussion

In this study, we performed the first cell-type informed EWAS of gingival tissue biopsies to identify CpG methylation differences between the uninflamed and periodontitis-inflamed oral mucosa. EWAS that investigate environment-induced epigenetic changes in tissues of multiple cell types need to address cell type deconvolution and intra-individual variation of methylation patterns. Likewise, we observed substantial immune cell infiltration within the inflamed gingiva leading to substantial effects on the methylome of the oral mucosa, according to which inflamed gingival tissue contained approximately twice as much infiltrated immune cells as clinically uninflamed tissue, with 52% in the total of 60 inflamed samples.

Surprisingly, we furthermore observed considerable amounts of immune cells in samples excised from periodontitis patients at sites diagnosed as uninflamed by specialized periodontologists, sometimes as high as in inflamed samples, with an average of 28% in the total of 60 clinically uninflamed biopsies sampled in this study. Compared to this, in a previous study, we identified immune cell proportions of 16% in oral masticatory mucosa samples from healthy individuals with no diagnosed oral inflammation, taken from the hard palate near the gingival margin [12]. It is conceivable that periodontitis patients show a general immune response in the affected tissue that leads to an increased infiltration of immune cells also at sites where no periodontal tissue inflammation was diagnosed. This underlines the importance of incorporating cell-type deconvolution algorithms in differential expression and methylation studies to avoid type I and type II errors.

Another limitation of EWAS is the complexity of intra-individual variation of methylation patterns, which has a stronger impact if sample size is limited. As a consequence, EWAS have higher genomic inflation factors compared to GWAS, where inter-individual genetic variation generally plays a much lesser role. Accordingly, the current study had a genomic inflation factor of 5.97 despite cell type deconvolution and removal of samples with ambiguous inflammatory data. We aimed to reduce intra-individual methylation variation by analyzing paired biopsies from 60 individuals on the EPIC BeadChip. However, after removing samples with an ambiguous immune state, our study comprised a non-paired analysis panel of 48 clinically uninflamed and 48 periodontitis-inflamed samples from 57 patients in total. Of these, 39 patients both donated an uninflamed and an inflamed biopsy. To increase statistical power and thus, to reduce the potential of false negative findings, the total sample panel of 2 × 48 non-paired samples was analyzed first. Subsequently, a sub-analysis of the significant results in the 2 × 39 paired samples was performed to remove potential false positive findings. This two-step approach in conjunction with cell type deconvolution, an improved intercept method for the estimation of confounder-informed effect sizes, recently developed by our group [16], and stringent filter criteria allowed us to identify several genes that showed robust different methylation between uninflamed and periodontitis-inflamed gingival tissues, independent of immune cell infiltration and inter-individual variation. Some of these genes have plausible roles in the etiology of periodontitis.

The most significant DMP with adjusted effect sizes mapped to the last intron of *PTP4A3* (Protein Tyrosine Phosphatase 4A3). *PTP4A3* has a role in the positive regulation of endothelium wound repair and angiogenesis [25] and inhibition of the expression of extracellular matrix and adhesion genes, like matrix metalloproteinases (MMPs) and integrins [26, 27]. Substrates of PTP4A3 are e.g. MMP14 [28], integrin β1 [27], and Keratin 8 (KRT8) [29]. The identification of *PTP4A3* is a good example of the suitability of our intercept method to provide effect sizes that directly correspond to the *p* value, which is missing in most EWAS. Currently, *p* values are usually based on log-transformed *M*-values because they exhibit better statistical properties, but are biologically meaningless. As a consequence, *p* values based on *M*-values are reported together with differences in raw *β*-values, which do not account for confounding factors. At the *PTP4A3* locus, such an approach would result in a highly significant *p* value with a ∆*β* of − 0.07 (calculated from the raw *β* = 0.87 in clinically uninflamed and 0.8 in inflamed tissue). The mean raw β at this locus is 0.8 in cultured GECs and 0.93 in blood cells. As the inflamed biopsies from the EWAS show an average amount of ~ 58% immune cells, 58% of the methylation signals in these samples are derived from cells that presumably exhibit methylation values as high as *β* = 0.93. Given the periodontal inflammation itself has no impact on methylation here, estimations for these highly admixed samples should be higher than for “pure” epithelial cells (i.e., the GECs). Instead, based on our intercept method, an adjusted ∆*β* of − 0.18 was estimated, indicating a strong hypomethylation of the gingival epithelial cells at this locus in inflamed samples.

The second most significant DMP mapped to the exonic region of *ROBO2* (Roundabout Guidance Receptor 2) that encodes a transmembrane receptor of the immunoglobulin superfamily and has been reported as a suggestive risk gene for periodontitis before [17]. It is involved in epithelial wound repair by promoting extrusion of dying cells from injured tissue [30]**.**

The highest adjusted effect size with a hypomethylation of 43% was observed in the gene *CCL26* (C–C Motif Chemokine Ligand 26). CCL26 is a factor for chemotactic eosinophil and basophil attraction in endothelial cells and possesses antimicrobial activity [31, 32]. Notably, *CCL26* was the most highly induced gene in patients suffering from eosinophilic esophagitis, a chronic allergic inflammation of the esophageal mucosa, compared with its expression level in healthy individuals [33].

Among the 20 genetic regions with the highest effect sizes was *PTP4A2* (Protein Tyrosine Phosphatase 4A2). This gene forms, together with *PTP4A3*, which harbored the most significant DMP in the full analysis as discussed above, and another phosphatase, *PTP4A1*, the subfamily of Phosphatase of Regenerative Liver (PRL). Given that two out of three PRL subfamily members showed highly significant DMPs in periodontitis-inflamed gingiva, the PRL subfamily represents a good candidate for evaluating its role in the inflammatory processes of oral epithelial cells.

In a more stringent filtering process, only “clusters” of DMPs that comprised at least 2 DMPs within 2 kb in the full analysis, at least one DMP with an effect size > 0.1, and one DMP significant in the paired analysis were included. In this way, further genes with a suggestive role in periodontal inflammation of the gingiva were identified. The most significant gene in the paired analysis, *DNAJC1* (DnaJ Heat Shock Protein Family Member C1), is an endoplasmatic reticulum heat shock protein that binds the molecular chaperone HSPA5 (alias BiP) [34], which is known to exhibit anti-inflammatory and inflammation-resolutory properties. BiP was proposed to form part of “resolution-associated molecular patterns” (RAMPs), intracellular responses to inflammatory and damage-induced stress, mediated by pathogen-associated (PAMPs) and damage-associated (DAMPs) molecular patterns [35]. Furthermore, BiP interacts with SERPINA3 [36], an acute phase protease inhibitor, which is supposed to inhibit angiotensin activation [18]. The second most significant DMP mapped to Leupaxin (*LPXN*), a focal adhesion protein, which showed a cluster of 6 DMPs. It is preferably expressed in hematopoietic cells with a putative role as an adapter protein in the formation of the adhesion zone in osteoclasts (Uniprot) and functions as a paxillin counterpart that potently suppresses the tyrosine phosphorylation of paxillin during integrin signaling [37]**.**

The DMP with the highest effect sizes from the stringent filtering approach mapped to *BPI* (Bactericidal Permeability Increasing Protein) and was also among the top 20 significant DMPs (paired analysis) in the stringent filtering approach. BPI is a lipopolysaccharide-binding protein with strong antimicrobial activity and an important part of innate immune response. It is predominantly expressed in hematopoietic cells, but is also found in a variety of other tissues, most notably the epithelial lining of mucous epithelial cells, where it was shown to block endotoxin-mediated signaling and to kill *Salmonella typhimurium* [38].

Enrichment analysis for the 2347 DMPs that were significant in the full as well as in the paired analysis showed no significant enriched GO terms or KEGG pathways after correction for multiple testing. However, GO terms with nominal *p* values < 0.001 were enriched for vesicle trafficking. Specifically, while the strongest enriched GO term, “inactivation of X chromosome by genetic imprinting”, encompassed only 3 genes, for the second most significant GO term, “synaptic vesicle cycle”, we found 25 out of 229 genes to be differentially methylated in periodontitis-inflamed gingiva. Likewise, the KEGG pathway “SNARE interactions in vesicular transport” was the 3^rd^ strongest nominally significantly enriched pathway (*p* = 0.036) and the functionally related pathway “Mucin type O-glycan biosynthesis” was the 8^th^ most enriched pathway out of 343 (*p* = 0.09). Interestingly, several genes among the most significant differentially methylated genes that were part of the vesicle trafficking gene sets are known to interact with the genes *VAMP3* and *VAMP8*, which are both suggestive risk genes of periodontitis [21–23]. *VAMP8* and *VAMP3* are highly expressed in keratinized oral mucosa, with TPM values of 2404 and 1839, respectively [12], indicating a functional role of these genes in epithelial and connective oral tissues. For example, in colonic epithelial cells, VAMP8 is required for the secretion of the main mucin of the mucin layer of the colon, Mucin-2, which has an important role in the maintenance of the mucosal barrier integrity. However, the observed trend of gene sets enriched for pathways of vesicle trafficking and mucin biosynthesis was not significant after correction for multiple testing, why these data need to be carefully interpreted.

In this context, a limitation of the present EWAS was the small sample size that impeded clear statistical evidence for enriched gene sets and a putatively more comprehensive set of DMPs. However, to our knowledge, it currently represents the largest EWAS that used gingival tissue biopsies and the only EWAS that analyzed paired inflamed and uninflamed samples from the same patients. Two previous EWAS that investigated differential methylation of periodontitis-inflamed gingiva [39, 40] included only 19 and 12 patients, respectively, and similar numbers of healthy controls. Moreover, the previous studies did not adjust for differences in cell type composition. Given the substantial immune cell infiltration, it is questionable whether findings from studies that ignore cell type deconvolution approaches point towards differential methylation patterns that reflect disease-relevant cellular processes or rather reflect differences in cell type composition.

In conclusion, this EWAS identified several genes that are significantly differentially methylated between periodontitis-inflamed compared to uninflamed gingiva. The strongest differences were observed for genes with roles in wound healing (*ROBO2, PTP4A3*), cell adhesion (*LPXN*) and innate immune response (*CCL26, DNAJC1, BPI*). Functional enrichment analyses implied that differentially methylated genes were overrepresented in gene sets annotated to vesicle trafficking. These results propose that the oral mucosa responds to a long-term inflammatory environment with specific adaptations in wound repair, barrier integrity, and innate host defense.

## Methods

### Study sample

A total of 60 periodontitis patients of Caucasian descent, aged 24–65, with ≥ 30% bone loss from the cement-enamel junction to the root apex, documented by a full-mouth set of radiographs or orthopantomographs, at ≥ 2 teeth were enrolled in this study. Written informed consent was obtained from all subjects according to the Declaration of Helsinki. All participants completed a detailed questionnaire to provide general personal information (e.g., sex, age, geographical and family descent), information on general and oral health, and smoking habits. This study was conducted in accordance with the principles of Good Clinical Practice and approved by the Independent Ethics Committee of each participating University Hospital.

### Collection of ex vivo tissue samples from clinically uninflamed and inflamed gingiva

Clinically uninflamed gingival tissue samples were collected during routine tooth extraction, surgical tooth lengthening, reopening of a submerged healing implant as anyhow performed during the intervention, with a tissue puncher with a diameter of 3 mm or from the tissue margins with a scalpel. A tissue sample of periodontitis-inflamed gingiva was taken from excised tissue due to modified Widman flap, osseous resective surgery [41], or with a disposable 3 mm gingival tissue puncher. To stabilize DNA and RNA, the biopsies were stored in the AllProtect reagent (Qiagen, Hilden, Germany) immediately after punching at 4 °C for 24 h, and subsequently transferred to − 20 °C.

### Cell culture of gingival epithelial cells and gingival fibroblasts

Solid ex vivo biopsies of the masticatory oral mucosa of the hard palate were taken from additional healthy subjects of Caucasian descent with 3 mm tissue punchers. Biopsies were dissected enzymatically to separate the epithelial cells from the fibroblasts by overnight incubation in 5 mg/mL dispase II (Sigma Aldrich) diluted in cell growth medium (DMEM, 1% Pen/Strep) at 4 °C. Immortalized and primary gingival fibroblast cells (iHGFs and pHGFs) were cultured in DMEM cell growth medium with 1% Amphotericin B, 1% Pen/Strep, and 1% non-essential amino acids. Immortalized gingival epithelial cells (OKG4) [42, 43], kindly provided by James Rheinwald, Boston, MA, USA, and primary GECs were cultured in DermaLife K medium with 1% penicillin/streptomycin in the presence of 60 μM or 1.4 mM Ca^2+^ (CellSystems, Troisdorf,Germany). Gingival fibroblasts and epithelial cells were cultivated in collagen-coated flasks at 37 °C and 5% CO_2_ until reaching 100% and 80% confluence, respectively, before DNA extraction.

### DNA extraction

The conserved ex vivo tissue samples were manually broken up into small pieces with a scalpel and subsequently homogenized using the automated mixer mill MM 400 (Retsch, Haan, Germany) with frozen beads (3 mm, Retsch) for 90 s at 30 Hz before further processing. DNA was extracted using the AllPrep DNA/RNA/miRNA Universal Kit (Qiagen, Hilden, Germany). DNA integrity was subsequently verified with a 2% agarose gel electrophoresis. Concentrations were measured using the Multiskan GO Microplate Spectrophotometer (Fisher Scientific, Hampton, USA). DNA samples were stored at − 80 °C.

### Bisulfite conversion and hybridisation to the Infinium MethylationEPIC BeadChips

500 ng DNA per sample was bisulfite converted with the EZ-96 DNA Methylation Kit (Zymo Research, Irvine, USA) and hybridized to the Infinium DNA MethylationEPIC BeadChip (Illumina, San Diego, USA) on an iScan Microarray Scanner (Illumina) at the Institute for Clinical Molecular Biology, Christian-Albrechts-University Kiel, Germany.

### Pre-processing and normalization

For analysis and quality control, the R environment (Version 4.0.3) and the R package minfi (Version 1.36.0) [44] were used, including the R package limma (Version 3.36.0) for the differential methylation analysis [45]. The Red/Green channel of the array were converted into one methylation signal without any normalisation. Using the function plotQC, sample-specific QC was estimated and no sample was removed. The QC criteria for probes were filtering of probes with median detection *p* values > 0.05, probes that lay within 5 bp of a single nucleotide polymorphism (SNP) with > 5% minor allele frequency, probes on the sex chromosomes and cross-reactive probes using the R package maxprobes (https://rdrr.io/github/markgene/maxprobes) according to Pidsley et al*.* [46] and McCartney et al*.* [47]. In total, 786,547 probes complied with the QC criteria and were included in the analysis. Methylation status was estimated according to the fluorescent intensity ratio, as any value between *β* = 0 (unmethylated) and 1 (completely methylated), and log2-transformed into *M*-values, which are considered a more statistically valid estimator [48]. After quality control, a functional normalisation was performed using the preprocessFunnorm function in minfi [49].

### Cell type deconvolution of EWAS samples

To identify the presence of non-epithelial cells in the oral mucosa samples, the EpiDISH algorithm (Version 2.6.0) [15] was used, applying the centEpiFibIC.m.rda reference dataset on our raw beta-values, which includes centroids for epithelial cells, fibroblasts, and immune cells (IC).

### Differential DNA methylation analysis

To identify DMPs correlating significantly with periodontal inflammation of the gingiva, we performed a linear mixed model (R package lme4, Version 1.1-27.1) [50] on the approximatively normally distributed *M*-values. We included affection status (inflamed vs. uninflamed) and IC-content (derived from the EpiDish algorithm) as fixed effects and the batch effect, i.e., slide and position on slide, as random effect with a constant intercept, using the R packages Combat and BatchQC [51]. Therefore, we adjusted the effect of the affection status, i.e., the delta *M*, for the confounder IC-content. Afterwards, we transformed the differences in *M*-values into differences in *β*-values by our recently developed intercept method [16]. By doing so, we were able to include the batch effect as well as the confounding effect of immune cell infiltration in gingival tissue.

To exclude potential stratification by the effects of smoking, which represents a major risk factor for periodontitis, 1501 probes that corresponded to DMPs in buccal mucosa of smokers were removed from the analysis beforehand [14]. Correction for multiple testing was carried out using the method by Benjamini and Hochberg [52]. CpGs were annotated to genes according to GRCh37/hg19 as provided in the MethylationEPIC BeadChip manifest.

In general, reported effect sizes Δβ were adjusted for IC-content and batch effects as described above, unless otherwise stated (i.e., referred to as “raw beta” values). To compare raw beta values of gingival cells with raw beta values of hematopoietic cells at significant DMPs, Illumina EPIC BeadChip data of 69 blood samples taken from 34 healthy female individuals at two different time points, was downloaded from the NCBI GEO database [53], accession GSE123914 [54].

### Stringent filtering approach

A more stringent filtering approach was performed for DMPs that were significant in the full analysis. As methylation is spatially correlated [55], next to the information on adjusted effect sizes, we incorporated information on nearby co-methylation in this filtering approach.The applied filtering criteria were: (1) ≥ 2 significant DMPs (*q* < 0.05 in the full analysis) had a maximum distance of 2 kb, (2) ≥ 1 DMP of such a cluster showed effect sizes > 0.1 in the full analysis, (3) ≥ 1 DMP of such a cluster showed *p*_adj_ < 0.05 in the paired analysis. We chose a maximum gap between DMPs of 2 kb as co-methylation was found to occur within this distance [56] and allowed for DMPs only associated in the full analysis as “supporting DMPs” to circumvent the problem of false negative findings in the paired analysis (which had less statistical power due to its limited size).

### Identification of differentially methylated regions

In addition to the stringent filtering process described above, which filtered also according to a spatial pattern of DMPs, we aimed to identify differentially methylated regions (DMR) using a distinct straightforward algorithm, tailored to find regions where methylation patterns deviate from the expected distribution. While this approach cannot account for our specific experimental setup with a subset of paired samples, it provides a high accuracy in finding true positive associations in “standard” case control settings, also controlling for confounding variables. To this end, we applied the Bump Hunter package, Version 1.32.0 [18], to our full dataset of 48 inflamed and 48 uninflamed samples and to the subset of paired samples. The cut-off value, which is a user-defined numeric value that determines the upper and lower bounds of the genomic profiles that are used as candidate regions, was set to 0.02 and the number of permutations was set to 1000.

### Gene ontology and gene set enrichment analysis of differentially methylated genes

For gene set and gene ontology enrichment analysis, we used the r packages missMethl and gometh [19]. The R package IlluminaHumanMethylationEPICanno.ilm10b4.hg19 (Version 3.13) was used for annotation. 344 KEGG terms were gathered using limma’s internal function getGeneKEGGLinks with default options for gene set enrichment analysis. 22,746 GO terms were derived fromorg.Hs.eg.db after cleaning. Gene ontology terms and gene sets with *q* < 0.05 were considered as being significantly enriched.

## Supplementary Information

**Additional file 1** (xlsx). EpiDish results for cell culture and biopsies. Given are the estimations for the proportions of the major cell types epithelial cells (Epi), fibroblasts (Fib), and immune cells (IC) per sample.

**Additional file 2** (png). Multidimensional Scaling (MDS) Plot for extraction sites. MDS Plot of the initial 120 samples, colored by site of extraction.

**Additional file 3** (xlsx). Significant DMPs in full set. Results for all 15,507 DMPs significant in the full set of 48 samples of clinically uninflamed and 48 samples of inflamed gingiva.

**Additional file 4** (xlsx). 441 DMPs from stringent filtering step. Results for the 441 DMPs in 193 clusters that were derived from a more stringent filtering process (*q* < 0.05, effect size ≥ 0.1, distance to nearest significant (*q* < 0.05) DMP $\le$ 2 kb, *p*_adj_ < 10^–5^ in paired analysis (*n* = 39) for ≥ 1 DMP in the cluster. TPM = transcripts per million, mRNA-sequencing in healthy keratinized oral mucosa with 16mio reads/sample [12].

**Additional file 5** (xlsx). Enrichment analysis of GO terms and KEGG pathways. Gene ontology (GO) terms and Kyoto Encyclopedia of Genes and Genomes (KEGG) pathways for genes corresponding to the 2347 DMPs significant in the full and paired sub-analysis. BP = biological process, MF = molecular function, CC = cellular component.

**Additional file 6** (png). Interaction networks of the 10 most significant genes from the GO term “synaptic vesicle cycle”. Interaction networks from the STRING database [20] for the top 10 differentially methylated genes in the GO term “synaptic vesicle cycle”.

## Data Availability

Methylation data from the EWAS experiment will be available from the corresponding author on reasonable request.
