# Supplementary figures and images for: Epigenetic adaptations of the masticatory mucosa to periodontal inflammation
Source: Clin Epigenetics. 2021 Nov 3;13:203. doi: 10.1186/s13148-021-01190-7 (PMC8567676; doi:10.1186/s13148-021-01190-7)

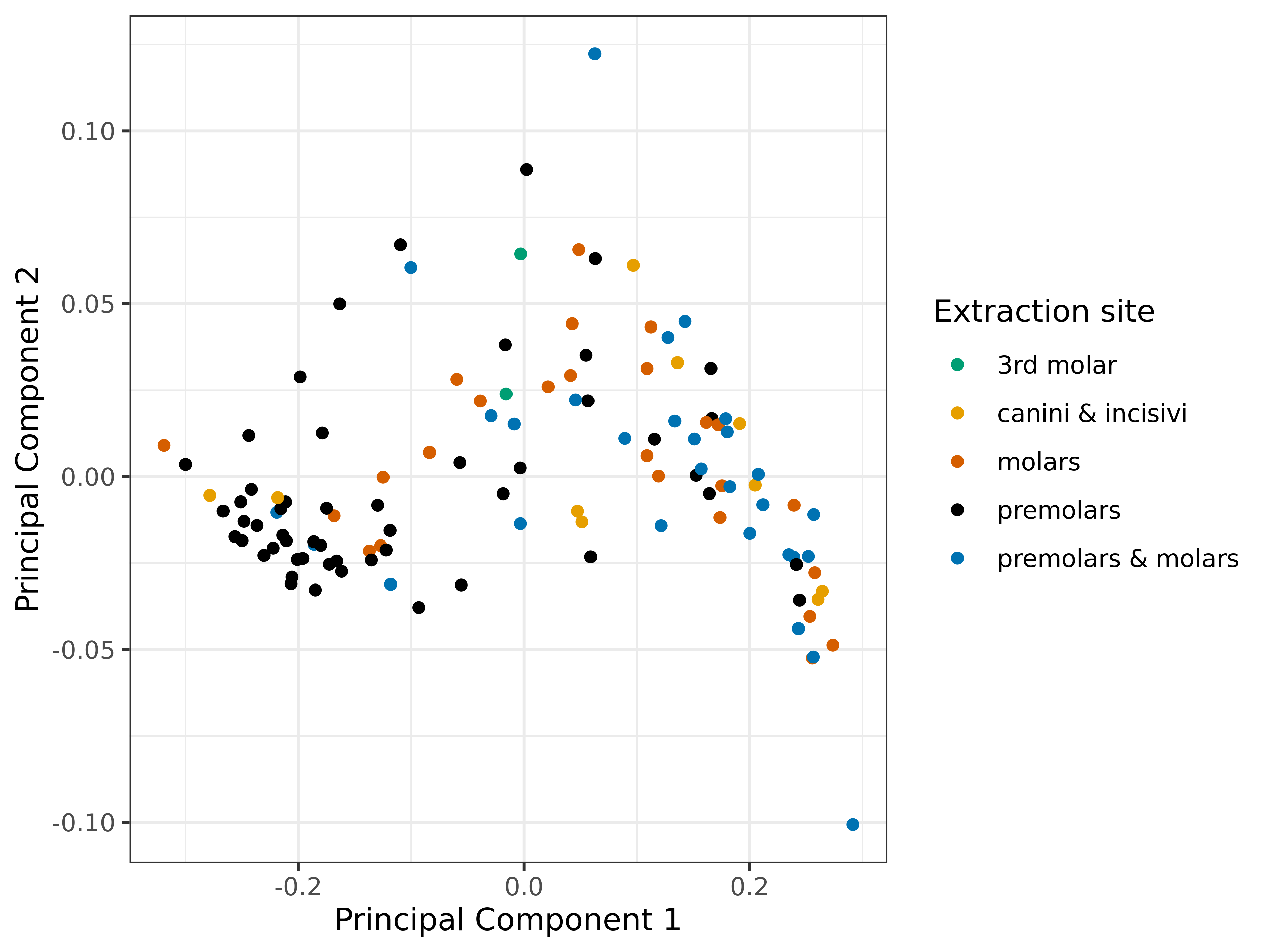

Supplement: Supplementary file 2 — Additional file 2 (png). Multidimensional Scaling (MDS) Plot for extraction sites. MDS Plot of the initial 120 samples, colored by site of extraction. [file 13148_2021_1190_MOESM2_ESM.png]

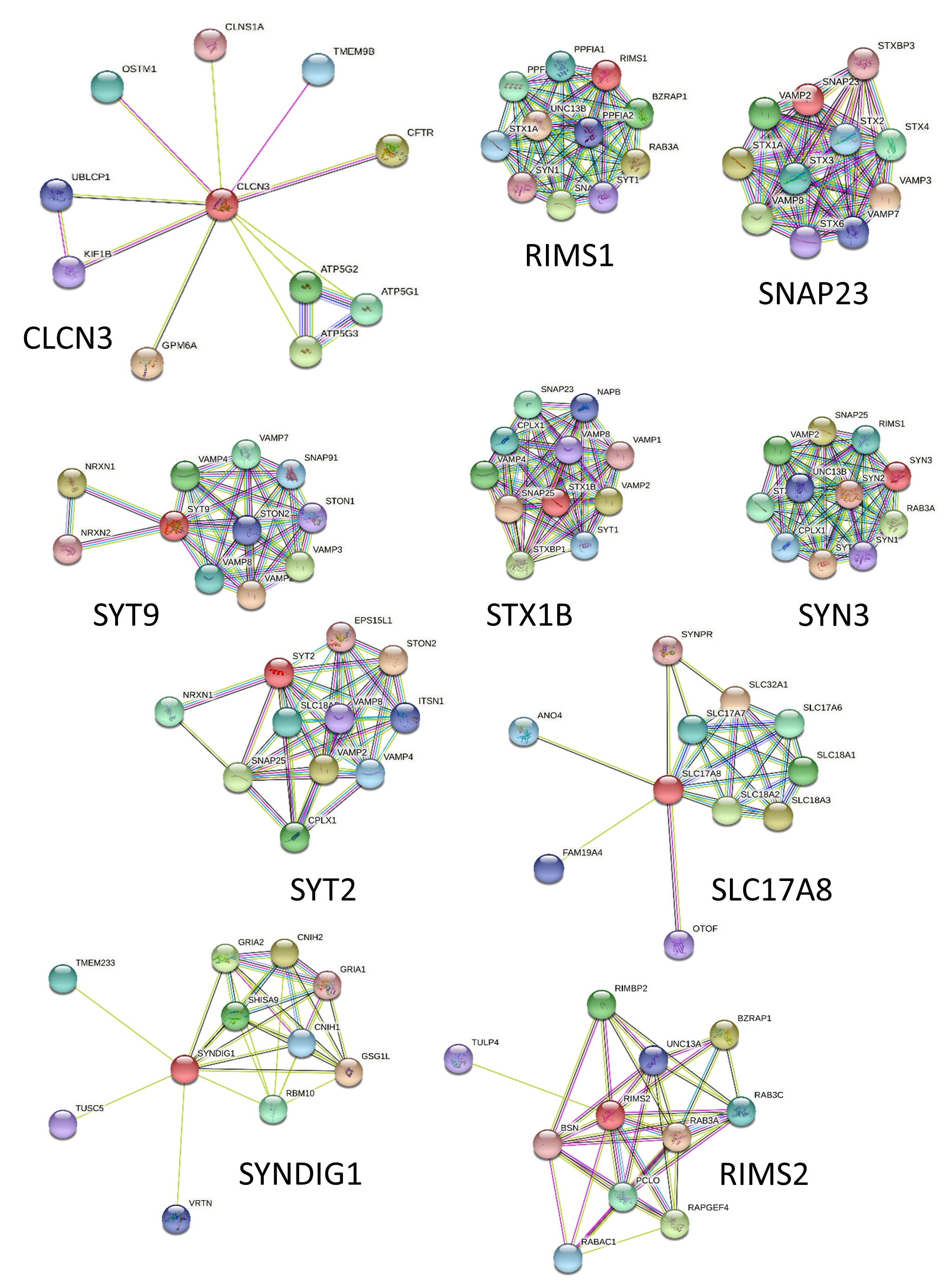

Supplement: Supplementary file 6 — Additional file 6 (png). Interaction networks of the 10 most significant genes from the GO term “synaptic vesicle cycle”. Interaction networks from the STRING database [20] for the top 10 differentially methylated genes in the GO term “synaptic vesicle cycle”. [file 13148_2021_1190_MOESM6_ESM.png]
